# Metabolomic, enzymatic, and histochemical analyzes of cassava roots during postharvest physiological deterioration

**DOI:** 10.1186/s13104-015-1580-3

**Published:** 2015-11-05

**Authors:** Virgílio Gavicho Uarrota, Marcelo Maraschin

**Affiliations:** Postgraduate Program in Biotechnology and Biosciences, Plant Morphogenesis and Biochemistry Laboratory, Biological Science Center, Federal University of Santa Catarina, Rodovia Admar Gonzaga 1346, Florianópolis, Santa Catarina CEP 88.034-000 Brazil

**Keywords:** Cassava, Deterioration, Metabolites, Enzymes, Histochemistry, Metabolomics, Chemometrics

## Abstract

**Background:**

Under postharvest physiological deterioration cassava root tubers alter the expression of biosynthetic pathways of certain primary and secondary metabolites, as well as the activity of some scavenging enzymes. Therefore, in this study we hypothesized that cassava cultivars differ as to their physiological responses to deterioration and their biochemical profiles can be an indicative of the tolerance or susceptibility to deterioration.

**Results:**

The results corroborate the working hypothesis, revealing that high Levels of phenolic acids, scopoletin, carotenoids, proteins, and augmented activities of guaiacol peroxidase and hydrogen peroxide in non-stored cassava roots can be used as potential biomarkers of cassava deterioration.

**Conclusions:**

Cassava physiological deterioration depends on cultivar and many compounds are up and downregulated during storage time. Secondary metabolites, enzymes, scopoletin, scavenging reactive oxygen species, and acidic polysaccharides are activated as responses to the physiological stress induced in root tubers.

**Electronic supplementary material:**

The online version of this article (doi:10.1186/s13104-015-1580-3) contains supplementary material, which is available to authorized users.

## Background

Cassava (*Manihot esculenta* Crantz) is the third most important source of calories and one of the major subsistence crops in the tropics, after rice and maize. Millions of people depend on cassava in Africa, Asia, and Latin America. Cassava ranks fifth among crops in global starch production [1]. It is grown by poor farmers, many of them women, often on marginal lands. For those people and their families, cassava is vital for both food security and income generation [2].

In rural areas of many cassavas growing countries the roots are mostly consumed fresh. As cassava harvesting can be staggered, rapid postharvest physiological deterioration (PPD) does not severely influence on-farm or village consumption [3]. In urban areas, unless motivated by economic considerations, consumers will not generally purchase old cassava roots (3–4 days after harvest) as they assume to be deteriorated. Cassava roots that exhibit visible symptoms of PPD are considered to have poor eating and processing quality, such as a longer time to cook, unpleasant bitter flavor, unattractive off color, lower and less desirable elasticity, and swelling of cooked byproducts [3, 4].

Fresh cassava roots are traditionally marketed without post-harvest treatment or protection and therefore have to reach the consumer within a very short time before deterioration becomes visible [5]. The negative effects of the rapid deterioration of fresh roots lead to high marketing margins. This particularly discourages consumption of cassava in urban areas where the roots have to compete with other foodstuffs.

Harvesting cassava is labor-intensive and its roots are bulky and highly perishable. Besides, far less research and technology have been devoted to cassava than to rice, maize, and wheat. This lack of scientific interest has contributed to highly uneven cultivation and processing methods, affording cassava products that often are of poor quality [2]. If more research is addressed in cassava deterioration, it could become the raw material base for an array of processed products that will effectively increase demand and contribute to agricultural transformation and economic growth in developing countries.

In this context, the present research aimed to investigate the changes in both primary and secondary metabolisms of four cassava cultivars under PPD using metabolomics and histochemical techniques. In a second approach, bioinformatics tools, i.e., data mining techniques were applied to the metabolomics data set towards better understanding the biochemical markers related to cassava PPD. Thus, we hypothesized that changes in metabolism and enzyme activities of the wound-induced deterioration (PPD) in cassava roots can serve as indicators of tolerance or susceptibility of genotypes to PPD.

## Methods

### Selection of cassava cultivars and on-farm trials

Four cultivars were selected for this study as follows: SCS 253 Sangão (hereinafter SAN), Branco (hereinafter BRA, a landrace), IAC576-70 (hereinafter IAC, a commercial variety), and Oriental (hereinafter ORI, a landrace). On-farm trials were carried out at the Ressacada Experimental Farm (Plant Science Center, Federal University of Santa Catarina, Florianópolis, SC, Brazil—27°35′48″ S, 48°32′57″ W), using the four cassava cultivars, as noted above, provided by Santa Catarina State Agricultural Research and Rural Extension Agency (EPAGRI) at Urussanga county (southern Brazil), the official state agriculture agency.

### Postharvest physiological deterioration (PPD)

Cassava root samples (12 months old) were collected for analysis of non-stored samples and for induction of physiological deterioration under controlled conditions in the laboratory. Immediately after harvest, the roots were washed, proximal and distal parts of the root were removed, and cross sections were made (0.5–1 cm) over the remaining root, followed by storage at room temperature (66–76 % humidity, 25 °C). Induction of PPD was performed for 11 days. Monitoring the evolution of PPD and associated metabolic disturbances were performed daily after induction of PPD. Non-stored samples and those at 3, 5, 8, and 11 days after PPD induction were collected at each time point, dried (35–40 °C/48 h) in an oven, milled with a coffee grinder (Model DGC-20N series), and kept for analysis. For enzymatic analysis, fresh samples were collected and stored (−80 °C) until analysis.

### Postharvest physiological deterioration scoring (PPD scoring)

Seven independent experiments of PPD were carried out in which a randomized sampling of 3 sliced roots from each plant variety was scored (from 1–10 % of PPD to 10–100 % of PPD) over the 11-day experimental period. The information was imaged through a digital camera (OLYMPUS FE-4020, 14 megapixel) and the results were analyzed by visual inspection of the images.

### Metabolomic, enzymatic, and histochemical analyzes

The dried and powdered cassava material (1 g per batch) was mixed with 10 mL ethanol 80 % and extracted using water bath at 55 °C, during 30 min. The mixture was centrifuged (4000 rpm/5 min), filtered on Whatman No. 2 filter paper, ethanol was removed using rotatory evaporator at 65 °C, and dried extract diluted to 3 mL with ethanol [6].

*The total phenolic* contents of the cassava extracts during PPD were determined through the Folin–Ciocalteau (FCR) method. For a 2.0 mL total volume, 200 µL of extract were first mixed with 100 µL FCR reagent after adding 1.40 mL distilled water and the contents were kept at room temperature for 10 min. Later, 300 µL Na_2_CO_3_ aqueous solution (20 %, w.v^−^) were added and incubated for 1 h. The absorbance was measured at 765 nm through a UV–visible spectrophotometer (Spectrumlab D180). Total phenolics content was expressed as µg of gallic acid equivalents/g of dry extract (µg GAE/g) using a standard curve (0–1000 μg/mL) of gallic acid [7].

*Carotenoid* content was determined according to the described method [8]. Briefly, 1 g of flour samples was added to 2 mL of cold acetone. After 10 min, 2 mL petroleum ether were added and mixed using ultraturrax for 1 min. Samples were then centrifuged (3000 rpm/10 min), supernatant collected, 2 mL sodium chloride 0.1 M were added, the solution centrifuged again (3000 rpm/7 min), dried in rotatory evaporator (55 °C), and the dried extract dissolved with 3 mL petroleum ether. Absorbance was read at 450 nm in a UV–visible spectrophotometer using the absorption coefficient of β-carotene in petroleum ether (2592 L/mol cm).

*For Anthocyanins*, 1 g of flour sample, 5 mL methanol acidified with 1 N HCl (85:15 v/v) were added and pH adjusted to 1. The solution was centrifuged (4000 rpm/15 min), the supernatant collected and dried in a rotatory evaporator (55 °C). The dried extract was reconstituted with 2 mL methanol and filtered (0.45 µm). Two dilutions were made, one to pH 1.0 buffer by using 3 M potassium chloride and other to pH 4.5 using 3 M sodium acetate buffer. Samples were diluted tenfold to a final volume of 2 mL and the absorbance read after 30 min of incubation at 520 and 700 nm (Spectrumlab D180 spectrophotometer) [9, 10].

*Total flavonoid* content of plant extract was determined using aluminum chloride colorimetric method [11, 12] and standard solutions (0–1000 µg/mL of quercetin in 80 % methanol). For that, 1 mL of extract solution was mixed with 0.5 mL 95 % ethanol (v/v), 0.1 mL 1 M potassium acetate, 0.1 mL aluminum chloride solution (10 % AlCl_3_), and 0.8 mL distilled water to a total volume of 2.5 mL. The mixture was well mixed and incubated at room temperature for 30 min, versus reagent blank containing water instead of sample. Quercetin was used as the standard (y = 0.0006x, r^2^ = 0.98) for the quantification of total flavonoid.

*To determine total cyanide*, the method reported by Bradbury [13] with some modifications was used. Briefly, 1 g flour samples during PPD were weighed out into plastic bottles; 10 mL 1 M phosphate buffer pH 7.0 and buffer paper were added. A picrate paper was also added; the bottle was closed with a lid and was left 16 h at 30 °C. The picrate paper was removed, eluted with 0.5 mL water, incubated during 30 min, and absorbance measured at 510. Acetone cyanohydrin was determined on the same flour samples as described for total cyanide, but by adding also 0.5 mL 0.1 M HCl.

*For the measurement of enzyme activity*, flour samples (1 g) from different days of PPD (0, 3, 5, 8, and 11) were homogenized in 5 mL 10 mM potassium phosphate buffer (pH 7.0) containing 4 % (w/v) PVP (Mr 25,000). The homogenate was centrifuged (4000 rpm/30 min) and the supernatant used as enzyme extract [14]. Catalase (CAT) activity was measured directly by the decomposition of H_2_O_2_ at 240 nm in a spectrophotometer (y = 2.1247x, r^2^ = 0.97) and expressed in units (U) per milligram (U mg^−1^, 1U = 1 mM of H_2_O_2_ reduced per minutes × milligrams of protein) [15]. The reaction mixture contained 1 mL 50 mM potassium phosphate buffer (pH 7.0), 1 mL 10 mM H_2_O_2_, and 1 mL of the extract. Hydrogen peroxide was determined according to Velikova [16]. 1 g flour sample was homogenized in ice bath with 5 mL 0.1 % (w/v) trichloroacetic acid (TCA). The homogenate was centrifuged (4000 rpm for 5 min), the supernatant collected (1 mL) and added of 50 mM 1 mL potassium phosphate buffer (pH 7.0) and 2 mL 1 M KI. The reaction mixture was read at 390 nm in a spectrophotometer and the content of hydrogen peroxide calculated through a standard curve (y = 2.1247x, r^2^ = 0.97).

*SOD family of enzymes* analysis was carried out according to Fridovich [17]. Briefly, 1 g flour sample was homogenized with 10 mL 50 mM potassium phosphate buffer (pH 7.0), centrifuged (4000 rpm/30 min) and the supernatant containing the crude enzyme extract for assay recovered. For total superoxide dismutase enzyme (Total SOD), 1 mL 0.05 M sodium carbonate buffer (pH 10.2) was added to 1 mL of enzyme extract and 0.5 mL 4 × 10^−4^ M of epinephrine. The rate of epinephrine auto-oxidation was determined by monitoring spectrophotometrically the absorbance in samples in a starting point of reaction and 2.5 min later. The MnSOD was assayed using the same method as above, except with the addition of sodium cyanide (NaCN), an inorganic compound with high affinity for metals to inhibit Cu/ZnSOD activity. The enzyme activity of Cu/ZnSOD was then determined as difference of total SOD and MnSOD

*The linamarin* solution was assayed in triplicate by adding 100 µL of the pink solution (previously described by Uarrota [18]) and 0.5 mL water to a small plastic bottle, followed by a 2.1 cm diameter filter paper disc previously loaded with phosphate buffer 0.1 M at pH 6 (3 mL) and 3 mL linamarase. A picrate paper was placed in the bottle, which was closed with a screw cap and left at 30 °C overnight. The brownish picrate paper was removed from the bottle and immersed in 5.0 mL water for 30 min and the absorbance of the solution measured at 510 nm (Spectrumlab D180 spectrophotometer). Linamarase assay was carried out by using 1.5 mL of the homogenate, 0.5 mL 5 mM linamarin in 50 mM of Na-citrate, pH 6.0 at 37 °C [19]. After 15 min, the reaction was stopped by boiling the reaction mixture for 2 min and the glucose released was measured using glucose oxidase method using glucose-oxidase kit (Glucose-PAP, LAB TEST diagonostica). Briefly, 3 mL kit reagent was added of 0.3 mL of sample, followed by mixing and incubation at 37 °C during 15 min and absorbance read at 520 nm (Spectrumlab D180 spectrophotometer). The glucose released in (mg/dL) was quantified and converted to mmol/L.

*For Polyphenol oxidase (PPO)* analysis, 2 g of fresh tissue were homogenized with 0.6 g of PVPP and 8 ml 50 mM (pH 7) phosphate buffer, recovering the supernatant by filtration and centrifugation (4000 rpm, 4 °C, 15 min, 18 cm of rotor radius) and this constituted a enzymatic extract. PPO activity was measured using 2.85 ml 0.2 mM (pH 7) phosphate buffer, 50 µl catechol (60 mM) as substrate, and 100 μl enzymatic extract, at 25 °C. Changes in absorbance (420 nm) were recorded over a 5 min period in a UV–visible spectrophotometer (Spectrumlab D180, BEL Photonics, Brazil—[20]).

*Ascorbic acid (AsA)* content was assayed as described previously with slight modifications [21]. The extract was prepared by grinding 1 g of sample with 5 ml 10 % TCA, centrifuged (3500 rpm, 20 min), re-extracted twice, and the supernatant made up to 10 ml and this constituted the extract. The reaction medium was done by 1.0 ml of extract, 1 ml DTC reagent (2, 4-dinitro phenyl hydrazine–thiourea–CuSO_4_), incubated (37 °C, 3 h) and 0.75 ml ice-cold 65 % H_2_SO_4_ (v/v) added after incubation, allowed to stand for 30 min, at 30 °C. The resulting color was read at 520 nm in the spectrophotometer (Spectrumlab D180, China). The AsA content was determined using a standard curve build with AsA (y = 0.0361x, r^2^ = 0.99, 0–1000 mg mL^−1^) and the results were expressed in µg g^−1^ (ppm) of fresh weight.

*Protein content* was determined in the cassava root samples (non-stored and 3, 5, 8, and 11 days postharvest) using Coomassie brilliant blue G-250 [22] reagent, with bovine serum albumin as standard (y = 0.0159x, r^2^ = 0.98). For enzymatic activities, cassava root samples (1 g, grated samples) were collected directly into liquid nitrogen in a mortar, with 2 % PVPP, 1 mM PMSF, 10 mM DTT, and 0.1 mM EDTA (MW: 292.2 g mol^−1^) in 50 mM Na-P buffer, pH 7.5. For analysis of ascorbate peroxidase (APX), the extraction buffer also contained 2 mM ascorbate (MW: 176.13 g mol^−1^). The suspension was centrifuged (4000 rpm, 30 min, 4 °C) and the supernatant used for enzyme assay.

*Ascorbate peroxidase* (APX) activity was measured by monitoring the decline in absorbance at 290 nm, as ascorbate (ε = 2.8 mM^−1^ cm^−1^) was oxidized, for 3 min [23]. The assay medium consisted of 1200µL 50 mM potassium phosphate buffer (pH 7.0), 200 µL EDTA, 200 µL ascorbate, 200 µL of sample, and 200 µl 0.1 mM H_2_O_2_ to start the reaction. APX activity was expressed in mM ascorbate min^−1^ mg^−1^ of proteins.

*Guaiacol Peroxidase* (GPX) activity was measured using a reaction medium containing 50 mM phosphate buffer (pH 7), 9 mM guaiacol, and 19 mM H_2_O_2_ [24]. The kinetic evolution of absorbance at 470 nm was measured during 1 min. Peroxidase activity was calculated using the extinction coefficient (26.6 mM^−1^ cm^−1^, at 470 nm). One unit of peroxidase was defined as the amount of enzyme that caused the formation of 1 mM tetraguaiacol per minute.

*Tocopherol* (α-TOC or vitamin E) activity was assayed as described by Backer [25] with small modifications. Briefly, 1 g of cassava sample was homogenized with 5 ml of a mixture of petroleum ether and ethanol (2: 1.6, v/v), the extract was centrifuged (4000 rpm, 30 min, 4 °C), and the supernatant was used to estimate α-TOC content. To one milliliter of extract, 3 ml 2 % 2, 2-dipyridyl in ethanol were added, mixed thoroughly, and kept in dark for 5 min. The resulting red color was diluted with 4 ml distilled water and mixed well. The resulting color in the aqueous layer was measured at 530 nm. The α-TOC content was calculated using a standard curve (y = 0.1115x, r^2^ = 0.96) of α-TOC (0–100 mg mL^−1^) and expressed in mg g^−1^ of fresh weight (FW).

*Sugars and organic acids* were extracted from 0.5 g of cassava root flour samples in 10 ml mobile phase (H_2_SO_4_, 5 mM) and determined accordingly [26]. Briefly, the suspension was homogenized using an ultra-turrax apparatus and mixed slowly using a horizontal shaker (Microplate shaker, 330 rpm), for 30 min. The suspension was centrifuged (8000 rpm, 10 min), filtered through a 0.22 μm disposable syringe membrane filter and the supernatant collected. Sugars and organic acids were analyzed by HPLC using a Biorad Aminex HPX 87H column, equipped with a UV detector (MWDG 1365D, for organic acids), connected in series with a refractive index detector (RID G 1362A, for sugars) and an injection valve fitted with a 15 μL loop. The samples were separated isocratically at 0.6 ml min^−1^ at 30 °C.

Retention times and standard curves were prepared for sugars and organic acids (see Additional file 1 : Table S1). Three consecutive injections (10 μL) were performed. Sugars and organic acids were expressed (mg g^−1^) as mean ± standard deviation.

*For scopoletin analysis*, cassava root flour samples (1 g) were placed in 50 mL falcon tubes containing 2 mL 98 % ethanol (JT Baker, USA) and homogenized with an ultraturrax (IKA T18 basic, IKA, China) for 30 s. The suspension was vortexed (1 min), incubated (microplate shaker, 600 rpm, 30 min), and centrifuged (7000 rpm, 5 min). The extract was filtered on a Whatman # 1 paper and through a 0.22 μm nylon membrane. Samples were transferred to 1.5 mL vials for HPLC (Agilent Technologies 1200 series, Waldbronn, Germany) analysis [27]. For that, samples (50 µL) were injected into an HPLC (Agilent Technologies 1200 series, Waldbronn, Germany), equipped with a reverse-phase column (Techsphere BDS C18, 250 mm × 4.6 mm, 5 μm) and a diode array detector. The column was kept at 25 °C and acetonitrile and 0.5 % phosphoric acid (v/v) in aqueous solution were used as mobile phase. The gradient profile was 60–1 % for 30 min with a 0.5 mL min^−1^ flow and 50 μL injection volume. Scopoletin was detected at 215, 280, and 350 nm and according to its retention time, using a standard compound sample (Sigma–Aldrich: scopoletin ≥99 %—no. S2500). Scopoletin quantification was determined through a calibration standard curve (y = 158159.59x, r^2^ = 0.99, 1–75 mg L^−1^). Three consecutive injections (10 μL) were performed and quantifications were made on a dry weight basis, and data represented in nmol g^−1^, as mean ± standard deviation.

*For histochemical analysis*, cassava root samples (non-stored and 3, 5, 8, and 11 days of PPD) were collected and small pieces were made (0.5 × 0.5 cm^2^) for subsequent fixation in paraformaldehyde. Samples of cassava roots were fixed in 2.5 % paraformaldehyde in 0.1 M (pH 7.2) phosphate buffer (72 h). Subsequently, the samples were dehydrated in increasing series of ethanol aqueous solutions [28, 29]. After dehydration, the samples were infiltrated with historesin (Leica Historesin, Heidelberg, Germany). Sections (5 μm length) were stained with different histochemical techniques and investigated with an Epifluorescent (Olympus BX 41) microscope equipped with Image Q Capture Pro 5.1 software (Qimaging Corporation, Austin, TX, USA). LM sections were stained as follows: Periodic Acid-Schiff (PAS) used to identify neutral polysaccharides [18], Toluidine Blue (TB-O) 0.5 %, pH 3.0 (Merck Darmstadt, Germany) used for acid polysaccharides through a metachromatic reaction [28], and Coomassie Brilliant Blue (CBB) 0.02 % (w/v) in Clarke’s solution (Serva, Heidelberg, Germany) used for protein identification [30].

### Statistical analysis

All statistical analyzes and graphics were implemented in R language (R core team-2014, version 3.1.2) [31]. Data are represented as mean ± standard deviation of a minimum of three repetitions (n = 3). Two-way ANOVA using randomized complete design was applied. Ordinary least square (OLS) regression models and decision regression trees were applied for predictive models (see Additional files 2, 3, 4, 5, 6, 7, 8 and 9). Histochemical micrographs were performed in Photoshop, version 7. Raw data in csv format—Additional files 2, 3 and 4, R software report (html format—Additional file 5) and data as R objects (RData format—Additional files 6, 7, 8 and 9) are also provided.

## Results and discussion

### Results

**Fig. 1:**
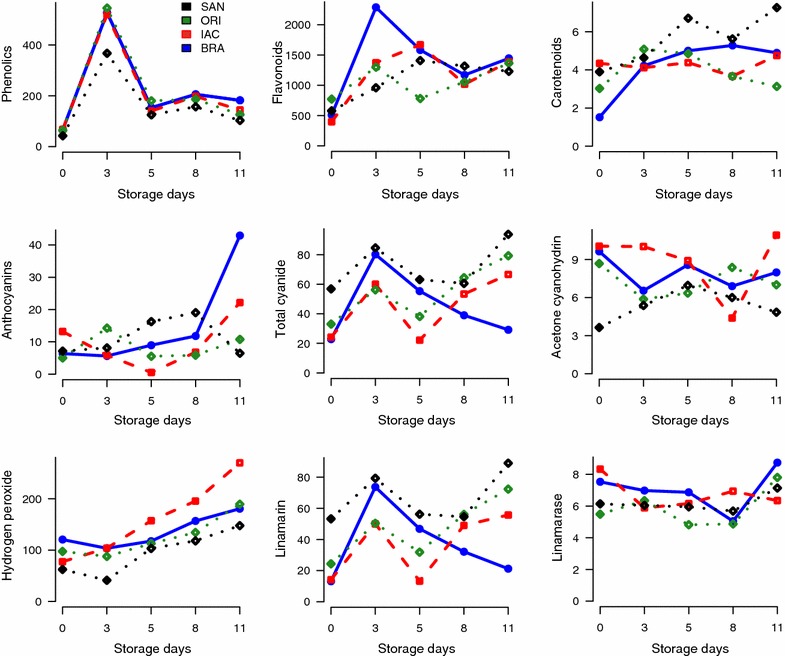
Changes in secondary metabolites (total phenolics, carotenoids, flavonoids and anthocyanins—μg/g), cyanogenic glucosides (total cyanide, linamarin, cetone cyanohydrin (mg/kg), and linamarase—mmol/L) and hydrogen peroxide (µg/g) in cassava cultivars during postharvest physiological deterioration (PPD). *Blue color* in the graphic represent Branco cultivar (BRA); *Red*-IAC576-70 (IAC); Forestgreen-Oriental (ORI), and *black*-Sangão (SAN). Values reported are means and standard deviations of a minimum of three repetitions

**Fig. 2:**
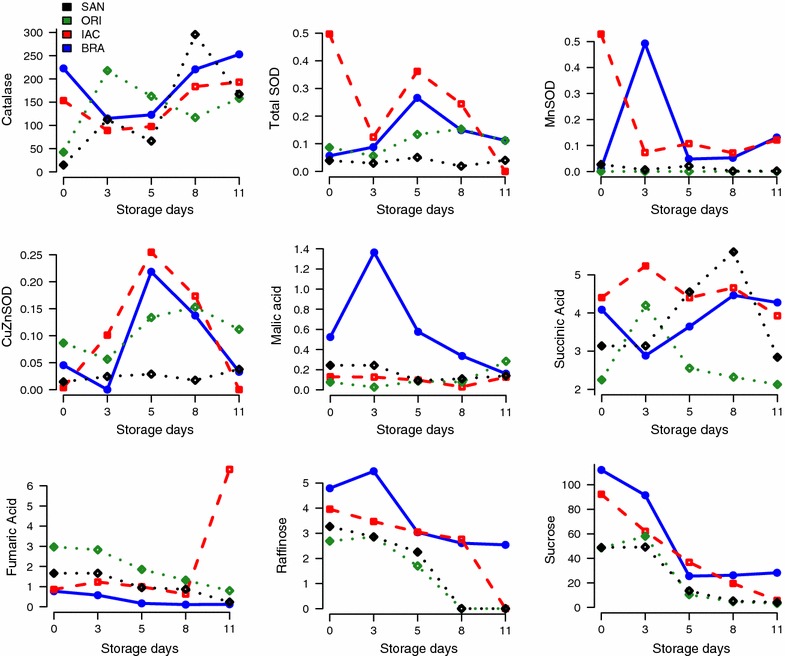
Changes in catalase (U/kg), SOD family of enzymes (U/kg), organic acids (malic, succinic, and fumaric—mg/g) and soluble sugars (raffinose and sucrose—mg/g) in cassava cultivars during postharvest physiological deterioration (PPD). *Blue color* in the graphic represent Branco cultivar (BRA); *Red*-IAC576-70 (IAC); Forestgreen-Oriental (ORI) and *black*-Sangão (SAN). Values reported are means and standard deviations of a minimum of three repetitions

**Fig. 3:**
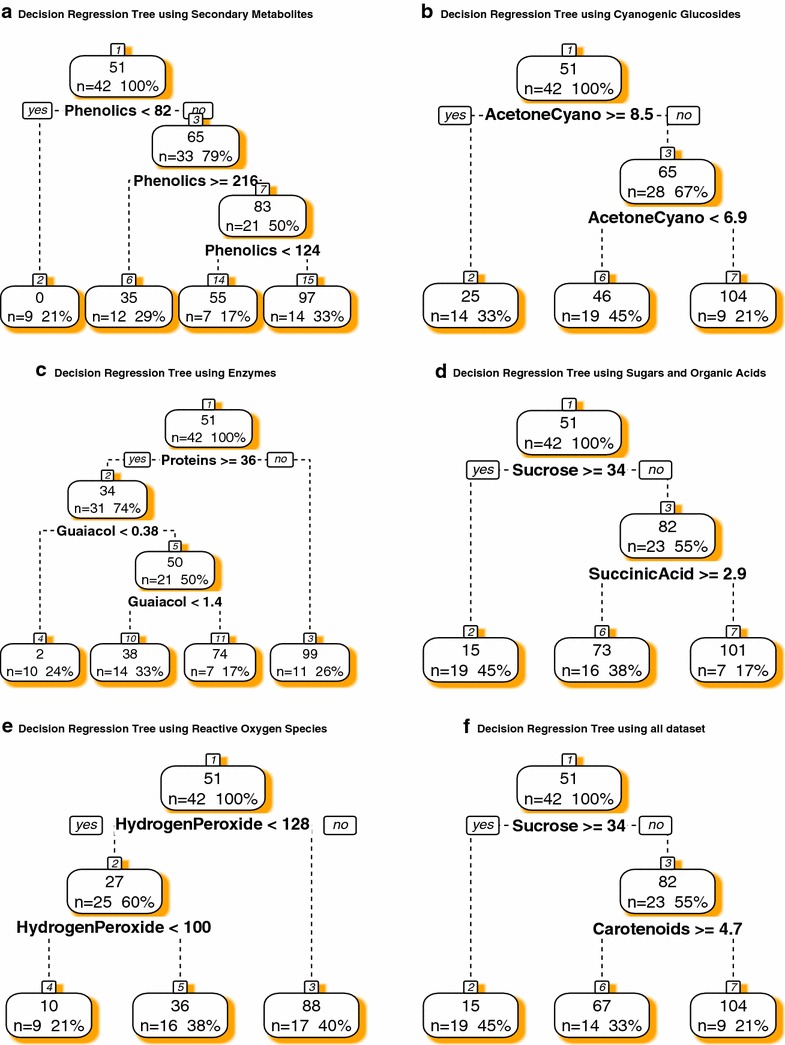
Decision regression trees showing the main compounds (predictors) related to PPD in cassava cultivars. Data were organized in small subsets to find the best model to predict PPD (**a** secondary metabolites, **b** cyanogenic glucosides, **c** enzymes, **d** sugars and organic acids, **e** reactive oxygen species and **f** all dataset containing 29 variables)

**Fig. 4:**
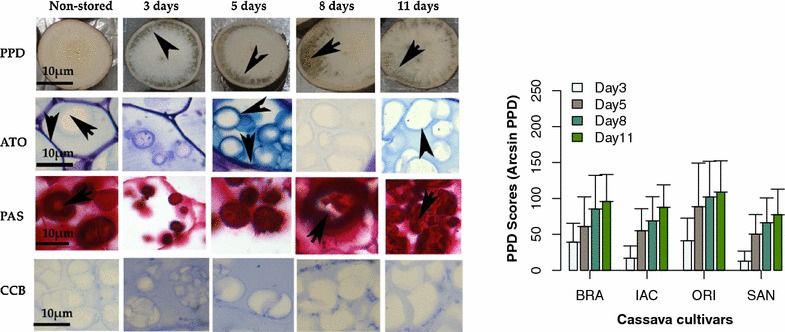
From *left figure* PPD induction images (PPD) from non-stored samples until 11 days of storage, Histochemical analysis (*ATO* Toluidine Blue, *PAS* periodic acid schiff, *CCB* Coomassie blue brilhant) of the susceptible cultivar ORI. *Right barplot* figure represents means and standard deviation scores of PPD analyzed in seven independent experiments with 3 repetitions each one

Total secondary metabolites (phenolics, flavonoids, carotenoids, and anthocyanins), cyanogenic glucosides (total cyanide, acetone cyanohydrin, linamarin, and linamarase), ROS (hydrogen peroxide), ROS-scavenging enzymes (CAT, total SOD, MnSOD, CuZnSOD, APX, GPX, PPO, Proteins), non-enzymatic antioxidants (AsA, α-TOC), soluble sugars, organic acids, and hydroxycoumarins (scopoletin) evaluated during storage time of cassava roots are summarized in Figs. 1, 2 and Table 1. Figure 3a–f shows the decision tree models with the main compounds related to PPD in cassava cultivars and Fig. 4 (left) summarizes the images derived from PPD induction of cassava roots (non-stored samples and those stored until 11 days) and histochemical analysis of samples stained with ATO, PAS and CBB. Figure 4 (right) represent the results of PPD scoring of cassava roots during the storage time (3, 5, 8, and 11 days). PPD rate increases during the storage time in all cultivars. Table 2 shows the results of ordinary least square (OLS) regression models of all data and subsets (secondary metabolites, cyanogenic glucosides, enzymes, sugar + organic acids, and ROS-scavenging enzymes).

**Table 1:** Changes in sugars (mg/g), scopoletin (mmol/g), polyphenol oxidase (U/mg min), ascorbic acid (µg/g), ascorbate (mM/min mg) and guaiacol peroxidase (μmol/min mg), tocopherol (mg/g), and proteins (mg/mL) during storage of cassava roots of four cultivars (SAN, ORI, IAC, and BRA)

Sample	Glucose	SD	Fructose	SD2	Scopoletin	SD3	PolyPhenol	SD4	Ascorbic	SD5	Ascorbate	SD6	Guaiacol	SD7	Tocopherol	SD8	Proteins	SD9
SAN	35.94c	1.8	26.06c	1.4	25.98d	2.1	4.57d	0.1	0.54d	0.0	1.32d	0.4	0.14d	0.0	5.58a	0.3	60.67a	0.1
SAN3	29.86d	0.8	26.78c	0.7	45.40c	0.8	6.16b	0.1	1.94bc	1.5	25.98b	0.2	1.36b	0.1	0.77d	0.3	66.89a	0.1
SAN5	67.11a	1.1	67.94c	1.2	120.80a	1.5	5.70c	0.1	1.42 cd	0.1	27.90a	0.3	0.52c	0.0	4.04b	0.5	82.82a	0.1
SAN8	55.60d	2.2	62.41b	2.6	125.81a	0.7	3.91e	0.1	3.57a	0.4	28.24a	0.9	1.27b	0.0	2.06c	0.5	65.74a	0.1
SAN11	18.14e	0.8	18.40d	0.8	66.64b	2.0	6.50a	0.2	2.67ab	0.1	7.07c	0.6	3.58a	0.3	0.42d	0.1	36.17a	0.1
ORI	15.48c	0.5	13.69c	0.5	18.59d	2.7	4.24b	0.3	0.64b	0.2	12.91d	1.7	0.19e	0.0	0.39b	0.1	39.32b	0.1
ORI3	30.23a	0.8	26.07b	0.4	124.89a	8.4	3.64c	0.1	0.73b	0.2	3.74e	0.8	1.34d	0.1	0.32b	0.1	55.82ab	0.1
ORI5	25.22b	0.2	31.62a	0.4	48.17c	1.0	4.26b	0.1	0.63b	0.4	20.20b	0.3	1.59c	0.0	0.23b	0.0	69.17ab	0.1
ORI8	17.09c	0.8	23.28b	1.2	81.80b	20.4	5.10a	0.1	1.82a	0.4	16.03c	0.5	5.07b	0.0	3.62a	0.1	21.55ab	0.1
ORI11	9.40d	0.1	13.41c	0.2	54.94c	0.8	3.82c	0.0	2.58a	0.0	118.99a	0.3	6.59a	0.2	0.25b	0.0	13.07a	0.1
IAC	82.26b	4.5	69.87c	4.3	64.25d	0.7	3.72c	0.1	0.29b	0.1	2.82e	0.5	0.14d	0.0	0.39b	0.1	43.60a	0.1
IAC3	45.74c	0.8	42.72d	0.9	81.81c	3.7	3.40d	0.1	1.25ab	0.0	34.32b	0.1	1.08c	0.1	2.11a	0.6	55.39a	0.1
IAC5	82.08b	2.8	84.25b	3.1	123.90b	2.2	4.54b	0.0	1.05ab	0.1	19.44c	0.2	0.24d	0.0	0.26b	0.1	110.53a	0.1
IAC8	91.77a	1.7a	99.80a	1.7	214.00a	0.3	2.98e	0.1	1.04ab	0.0	7.83d	0.3	1.84b	0.1	0.36b	0.1	57.46a	0.1
IAC11	42.23c	0.9	44.65d	0.7	98.10c	2.0	5.04a	0.1	1.33a	0.4	73.73a	1.8	3.45a	0.1	0.35b	0.1	18.24a	0.1
BRA	55.91d	2.1	54.19e	2.1	91.46c	7.6	4.19b	0.4	0.63b	0.5	14.01d	0.3	0.22d	0.0	0.21b	0.0	37.39a	0.1
BRA3	87.30c	1.2	81.25d	1.1	92.11c	3.5	3.21d	0.0	2.36a	0.2	31.05b	0.5	0.77c	0.0	0.25b	0.1	56.24a	0.1
BRA5	117.85a	1.1	125.74a	0.6	95.00c	9.5	3.64c	0.1	2.43a	0.3	44.63a	0.2	0.83c	0.0	0.23b	0.0	52.60a	0.1
BRA8	102.35b	2.0	117.27b	2.3	193.96b	18.2	3.70c	0.0	2.64a	0.2	26.46c	1.1	3.11a	0.1	0.25b	0.0	80.04a	0.1
BRA11	88.16c	5.0	111.09c	6.2	223.08a	5.0	6.53a	0.1	3.20a	0.3	9.04e	0.6	1.17b	0.1	2.56a	0.7	27.32a	0.1

Values are represented by means of a minimum of three repetitions (n = 3) followed by standard deviations (SD) of the compounds mentioned above (SD, SD2, SD3, SD4, SD5, SD6, SD7, SD8, and SD9) respectively. Numbers after cultivar name (e.g., SAN3) mean days of storage. Different letters in the columns and for each cultivar mean significant statistical differences during storage period for that cultivar (p < 0.05, Tukey HSD test)

**Table 2:** Results of ordinary least square (OLS) regression models tested using different subsets of data (metabolites, cyanogenic compounds, enzymes, sugars and organic acids, and ROS-scavenging enzymes)

	Dependent variable: PPD scores					
	Metabolites	Cyanogenics	Enzymes	Sugar + Acids	ROS^a^	All data
OLS regression models						
Constant	2.2 (20.5)	13.9 (51.7)	−13.2 (28.3)	92.9*** (19.2)	−16.8 (15.4)	−191.1 (221.9)
Phenolics	−0.1** (0.04)					−0.1 (0.1)
Flavonoids	0.04** (0.01)					0.01 (0.03)
Carotenoids	0.3 (3.9)					−1.9 (5.1)
Anthocyanins	0.4 (0.7)					0.9 (1.5)
Scopoletin	0.2 (0.1)					0.4 (0.4)
Total cyanide		16,723.7 (14,490.4)				20,536.5* (11,608.2)
AcetoneCyano		−16,721.6 (14,489.8)				−20,516.5* (11,607.2)
Linamarin		−16,723.2 (14,490.3)				−20,535.1* (11,608.2)
Linamarase		−0.3 (5.8)				12.6 (24.7)
Polyphenol			1.5 (5.1)			−18.9 (20.8)
Ascorbic			10.2* (5.2)			8.4 (11.7)
Ascorbate			−0.1 (0.2)			−0.7 (0.9)
Guaiacol			16.2*** (4.0)			11.7 (14.7)
Tocopherol			−0.5 (3.3)			5.1 (6.4)
Proteins			0.3 (0.2)			1.0 (1.3)
Malic acid				18.7 (18.2)		20.5 (53.7)
Succinic acid				−6.9 (5.7)		−20.7 (14.0)
FumaricAcid				−0.1 (3.1)		4.8 (13.0)
Raffinose				−15.7** (7.5)		40.2 (35.5)
Sucrose				−2.4** (1.0)		−4.1** (1.8)
Glucose				−4.0*** (1.3)		−2.6 (5.3)
Fructose				−0.3 (1.2)		−2.2 (5.1)
Total sugars				2.3** (0.9)		1.8* (1.0)
Hydrogen peroxide					0.4*** (0.1)	−0.1 (0.4)
Catalase					0.1 (0.1)	0.4 (0.2)
Total SOD					−56.7 (70.3)	100.8 (154.5)
MnSOD					−3.6 (53.6)	−28.9 (118.2)
CuZnSOD					143.3 (98.4)	−50.2 (177.6)
Observations	60	60	60	60	60	60
Log likelihood	−306.7	−312.9	−298.1	−288.0	−300.4	−274.3
Akaike Inf. Crit.	625.4	635.8	610.1	594.0	612.7	606.5

Coefficients of the generalized linear models (GLM) are presented and in parenthesis standard deviations are shown taking PPD as target to build the models. AIC is also presented for each model tested

Significance levels: * p < 0.1; ** p < 0.05; *** p < 0.01

^a^ROS—scavenging enzymes

### Discussion 

The plants have developed defense mechanisms to protect them from various disturbances (e.g., PPD induction). In addition to the constitutive barriers, plants acquire tolerance and resistance to various biotic and abiotic factors, due to its ability to activate defense mechanisms such as hypersensitivity responses, strengthening of the cell wall, oxidative species scavenging, and production of secondary metabolites [32].

Cassava samples showed significant increase in contents of phenolics, flavonoids, total cyanide, and linamarin during the first 72 h of storage, thereafter, a significant decline was observed (p < 0.05). Hydrogen peroxide showed a different trend as it continued increasing during the storage time. Linamarase activity and acetone cyanohydrin showed a slight decline (Fig. 1). The oxidative stress during PPD can damage cellular components such as DNA, lipids, proteins, and sugars. To minimize stress-related damage, the ROS homeostasis in plants is a complex process and phenolics, and flavonoids were reported to act in this process [33]. Cyanogenic glucosides such as total cyanide and linamarin can modulate oxidative stress and as phytoanticipins, they are being regarded as constitutive defense system during PPD [34, 35].

Catalase declined in BRA and IAC cultivars until 72 h and then increased in all cultivars. Total superoxide dismutase (SOD) increased until 110 h except for IAC cultivar, where a decline until 72 h was observed. Increases in manganese SOD were observed only for BRA cultivar, while for copper/zinc SOD increases were observed for BRA, ORI, and IAC until day 5 of storage (Fig. 2). A common feature of several types of ROS is their ability to cause oxidative damage to DNA, proteins, and lipids. These cytotoxic properties of ROS explain the evolution of complex mechanisms of enzymatic and non-enzymatic detoxification in plants. Under physiological conditions of stress such as PPD, ROS are scavenged by antioxidant systems confined in different cellular compartments. This explains the changes observed in CAT, hydrogen peroxide, and SOD family of enzymes in cassava roots.

The main organic acids found by high performance liquid chromatography (HPLC) were succinic, fumaric, and malic acids. A decline in fumaric and malic acids amounts was found, except for BRA who showed increases in malic acid until 72 h of storage. For succinic acid, an oscillation was observed in all cultivars studied. Organic acids are key components in response to nutritional deficiencies, metal ion accumulation, and plant–microorganism interaction. They can enhance resistance to diseases and inhibit oxidation during storage at low temperature, which significantly extends the storage life of plant biomasses [36]. They have also been related to the maintenance of membrane integrity in stress conditions [37].

The main soluble sugars found by HPLC were raffinose, sucrose, fructose, and glucose (Fig. 2; Table 1). Raffinose and sucrose declined during the storage time, while glucose and fructose showed different trends, i.e., an increase of glucose from 72 h (day 3) to day 5 of storage, except for SAN and IAC where a small decrease was found 72 h of storage. Fructose levels increased in all cultivars except for IAC where a slight decrease was observed 72 h of storage (Table 1). Cassava starch can be converted to maltotriose, maltose, and glucose, as well as to other modified sugars and organic acids [38]. Many studies have been devoted to soluble sugars metabolism in crop species, e.g., hexoses, sucrose, and maltose of stored yam tubers increased greatly during the storage period, starch-sucrose inter-conversion occurs during tuber storage. This finding explain increases of some sugars observed (e.g., glucose) during PPD.

Scopoletin increased in all cultivars until 192 h of storage (day 8) except for ORI where an oscillation was found. Polyphenol oxidase (PPO) showed a small decrease 72 h of storage and thereafter continued increasing during storage. Ascorbic acid did not show a typical trend during storage. Ascorbate and guaiacol peroxidases revealed a similar trend in general, increasing during storage time. Importantly, in all cases herein reported the changes were cultivar-specific. BRA cultivar showed to be more tolerant to postharvest physiological deterioration (PPD) and ORI the most susceptible. The formation of scopoletin occurs immediately after the rupture of the root tissues during the harvesting of cassava, with strong evidence that this metabolite contributes for deterioration of cassava roots [39, 40]. Increases in APX, GPX, AsA, and CAT subsequently detoxify the hydrogen peroxide in cassava roots during PPD.

Aiming at to understand which variables are mainly related to PPD, OLS-regression models were built for all dataset and subsets which included: enzymes, secondary metabolites, cyanogenic glucosides, enzymes, and ROS-scavenging enzymes (Table 2). Using a subset of data of secondary metabolites, we found that phenolics (negatively) and flavonoids (positively) significantly correlated to PPD (p < 0.05). Guaiacol peroxidase (p < 0.01) and ascorbic acid (p < 0.01) were the main predictors correlated positively to PPD using a subset data of enzymes (Table 2). By using sugars and organic acids as a subset of data it was found that raffinose, sucrose (p < 0.05), and glucose (p < 0.01) negatively correlated to PPD. Hydrogen peroxide was positively correlated to PPD (p < 0.01) in a subset data of ROS-scavenging enzymes. Using all dataset, we found that total cyanide is positively correlated to PPD while linamarin and acetone cyanohydrin are negatively correlated to PPD. When all models were compared using Akaike Information Criterion (AIC), we found that the best predictive model was that of sugars and organic acids, but AIC did not differed significantly in all models (Table 2).

When decision regression trees (Fig. 3a–f) were applied to the data (subsets and all data combined) aiming at to find biochemical markers related to PPD or best predictors for that physiological disturbance, it was found that phenolic compounds (Fig. 3a), acetone cyanohydrin (Fig. 3b), proteins, guaiacol peroxidase (Fig. 3c), sucrose, succinic acid (Fig. 3d), hydrogen peroxide (Fig. 3e), carotenoids, and sucrose (Fig. 3f) as most correlated to PPD.

Cassava samples at different storage days were also stained with toluidine blue (TB), periodic acid schiff (PAS), and Coomassie brilliant blue (CBB). The results of the claimed susceptible cultivar ORI are summarized in Fig. 4. In the same figure (right side) are also presented results of PPD scoring in all cultivars studied and micrographs of root slices of ORI cultivar during PPD. In general, all cultivars showed metachromatic reaction in the cell walls and around starch granules. This reaction was predominantly observed up to 5 days of storage, while for other cultivars it was detected only in the cell walls. Metachromatic reaction indicates the presence of acidic polysaccharides that are eventually produced as oxidative stress increases [41] in cassava samples, and their role can be attributed to reducing of the PPD stress. The degradation of starch granules could also be observed during storage. Samples stained with periodic acid schiff (PAS) exhibited a strong reaction for starch granules. A strong reaction is indicative of a major presence of neutral polysaccharides (i.e., starch) in these samples. Starch granules can be clearly observed in non-stored samples, while their degradation is clearly visible during storage. Starch is probably degraded during storage to form free sugars (e.g., glucose). When samples of cassava were stained by Coomassie brilliant blue (CBB), a slight reaction was found up to day 3 of storage in all samples for cell walls and around starch granules (Fig. 4). The reaction was more intense in BRA/SAN cultivars. These results corroborate the findings of protein quantification, which showed small increases in protein amounts from day 3 to 5 of storage. In general, cassava root samples are recognized to be poor in protein content, which explains the small reaction observed in all samples.

## Conclusions

On the basis of the results presented herein and previous studies published [3, 42–47], PPD in cassava roots depends on cultivar and many compounds are up and downregulated during storage time. Secondary metabolites, scopoletin (hydroxycoumarin), ROS-scavenging enzymes, and acidic polysaccharides are activated as responses to the physiological stress induced in root tubers. These compounds seem to play an important role in reducing or delaying the physiological deterioration process.

Sugars and organic acids are formed as result of starch degradation during storage and also the presence of organic acids can be an indicative of microbiological PPD. High contents of phenolic acids, scopoletin, proteins, carotenoids, and hydrogen peroxide, as well as the increase of the guaiacol peroxidase activity in non-stored cassava roots can be used as potential biomarkers related to the tolerance of PPD. The main hydroxycoumarin identified in cassava was scopoletin as the more tolerant cultivars to PPD showed higher amounts of this metabolite. The contents of scopoletin were increased during PPD, suggesting that scopoletin must be involved in reducing deterioration rate at the initial stage of PPD. The disruption of cellular compartments derived from tissue injury at the time of harvest allowed linamarase to get in contact with its substrate limanarin, a fact verified by the increase of the levels of hydrocyanic acid up to 3–5 days of storage followed by degradation of that metabolite. Linamarase activity was elevated in these stages (3–5 days). However, taking into account that PPD can also be related to abiotic factors and to genotypes (as herein shown), additional researches should be carried out in order to better understand why cassava deteriorate soon after harvest. ORI cultivar was the more susceptible cultivar to deterioration. GPX and APX activities and total proteins are increased during PPD. Similarly, antioxidant mechanisms also take place to ameliorate ROS production during this particular stress condition, helping to delay PPD. Histochemical analysis demonstrated that acidic polysaccharides seem to act as barrier components of plant cell walls and may play an important role in PPD delay as starch catabolism is observed during PPD. Finally, the pattern recognition models (supervised model) proposed was capable of classifying samples according to their metabolic profiles and degree of deterioration.

## Availability of supporting data

The data sets supporting the results of this article and scripts used for data mining in R language are made available as Additional file 9 in form of html report. Additional file 1: Table S1 is also provided.

## Additional files

10.1186/s13104-015-1580-3 HPLC standard curves prepared for sugars and organic acids studied. Three consecutive injections (10 ul) were perfomed. Sugars and Organic acids were expressed in (mg/g) as mean +/− standard deviation.
10.1186/s13104-015-1580-3 Raw data (in csv format) of cyanogenic glucosides used for decision tree model of Fig. 3a.
10.1186/s13104-015-1580-3 Raw data (in csv format) of enzymes used for decision tree model of Fig. 3b.
10.1186/s13104-015-1580-3 Raw data (in RData format) used PPD scoring of cassava cultivars during storage (Fig. 4-Right).
10.1186/s13104-015-1580-3 An html report of all statistical analyses conducted in the manuscript and produced in R Software (version 3.2.2).
10.1186/s13104-015-1580-3 Raw data (csv format) of scavengers of reactive oxygen species used for decision tree model of Fig. 3c.
10.1186/s13104-015-1580-3 Raw data (csv format) of secondary metabolites used for decision tree model of Fig. 3d.
10.1186/s13104-015-1580-3 Raw data (csv format) of sugars and organic acids used for decision tree model of Fig. 3e.
10.1186/s13104-015-1580-3 Raw data (RData format) of all compounds studied in this manuscript (cyanogenics, enzymes, ROS-scavengers, secondary metabolites, sugars and organic acids) used for decision tree model of Fig. 3f, two-way ANOVA and OLS-regression models.

## Abbreviations

PPD: postharvest physiological deterioration
ROS: reactive oxygen species
SOD: superoxide dismutase
