# Supplementary material for: Metabolomic, enzymatic, and histochemical analyzes of cassava roots during postharvest physiological deterioration
Source: BMC Res Notes. 2015 Nov 5;8:648. doi: 10.1186/s13104-015-1580-3 (PMC4634721; doi:10.1186/s13104-015-1580-3)
Supplement: Supplementary file 1 — 10.1186/s13104-015-1580-3 HPLC standard curves prepared for sugars and organic acids studied. Three consecutive injections (10 ul) were perfomed. Sugars and Organic acids were expressed in (mg/g) as mean +/− standard deviation. [file 13104_2015_1580_MOESM1_ESM.doc]

Supplementary data

Table captions

**Table 1**. HPLC standard curves prepared for the sugars and organic acids studied. Three consecutive injections (10 μl) were performed. Sugars and organic acids were expressed (mg g^-1^) as mean ± standard deviation.

| **Group of compound** | **^*^Compound** | **Code** | **Standard curve** | **r^2^** |
| --- | --- | --- | --- | --- |
|  | Glucose | G7528 | y=26748656x-1523663 | 0.99 |
| Soluble Sugars | Fructose | F2543 | y=26028204x-8253663 | 0.99 |
|  | Raffinose | [R0514](http://www.sigmaaldrich.com/catalog/product/sigma/r0514?lang=pt&region=BR) | y=22680182x+45255.3 | 0.99 |
|  | Sucrose | S7903 | y=22582989x+727997.7 | 0.99 |
|  | Citric | CO759 | y=3281.1x+46046 | 0.99 |
| Organic acids | Malic | 240179 | y=2498.2x+3816.4 | 0.99 |
|  | Succinic | S3674 | y=1737.8x-4255.3 | 0.99 |
|  | Fumaric | R412205 | y=4047.85x-5748.3 | 0.99 |

^*^All reagents were acquired from Sigma-Aldrich
