# Supplementary material for: Metabolomic, enzymatic, and histochemical analyzes of cassava roots during postharvest physiological deterioration
Source: BMC Res Notes. 2015 Nov 5;8:648. doi: 10.1186/s13104-015-1580-3 (PMC4634721; doi:10.1186/s13104-015-1580-3)
Supplement: Supplementary file 5 — 10.1186/s13104-015-1580-3 An html report of all statistical analyses conducted in the manuscript and produced in R Software (version 3.2.2). [file 13104_2015_1580_MOESM5_ESM.html]

### Title: Metabolomic, Enzymatic, and Histochemical Analyzes of Cassava Roots during Postharvest Physiological Deterioration

### Authors: “Uarrota VG, Maraschin M (2015)”

### Supporting data and scripts used in data mining for the results reported in the manuscript

```
setwd("C:/Users/Virgílio/Desktop/PASTAS DO DESKTOP/ascorbato peroxidase")
require(reshape)
require(reshape2)
load("MediasTabelaFinal.RData")  ##load the data "means3"
means3
```

```
##    Sample  Days Phenolics Flavonoids Carotenoids Anthocyanins Totalcyanide
## 20    SAN Fresh  42.57667   578.6110    3.900333     7.124667     56.86633
## 17    SAN  Day3 367.29667   959.1667    4.633333     8.126667     84.65167
## 18    SAN  Day5 125.16000  1408.0557    6.709000    16.253667     63.15733
## 19    SAN  Day8 156.85667  1317.4997    5.625000    19.036667     60.56767
## 16    SAN Day11 103.00000  1230.8333    7.268333     6.457000     93.79167
## 15    ORI Fresh  64.01000   772.5000    3.025000     5.010000     33.04100
## 12    ORI  Day3 545.22000  1299.1663    5.084667    14.249667     56.21133
## 13    ORI  Day5 180.17000   781.3890    4.853333     5.511000     38.15967
## 14    ORI  Day8 186.54000  1054.7223    3.661333     5.844667     64.45233
## 11    ORI Day11 125.91333  1365.2777    3.132667    10.742667     79.32000
## 10    IAC Fresh  67.73667   398.0553    4.344333    13.192000     24.20567
## 7     IAC  Day3 519.27667  1373.6113    4.116333     5.844667     60.04967
## 8     IAC  Day5 140.40000  1670.2780    4.379000     0.501000     22.18000
## 9     IAC  Day8 198.03000  1018.0553    3.673000     6.791000     53.37767
## 6     IAC Day11 144.13000  1386.9447    4.753333    22.154000     66.61533
## 5     BRA Fresh  66.79333   509.1663    1.516000     6.345333     22.80433
## 2     BRA  Day3 527.02333  2288.0553    4.216667     5.622000     80.21867
## 3     BRA  Day5 153.39667  1583.6110    5.004000     8.961333     55.37300
## 4     BRA  Day8 205.72667  1170.2777    5.281667    11.800333     39.02800
## 1     BRA Day11 182.07667  1445.2777    4.895667    42.916000     29.17200
##    AcetoneCyano Linamarin Linamarase HydrogenPeroxide  Catalase   TotalSOD
## 20     3.640667  53.22533   6.135333         62.26767  14.88867 0.03933333
## 17     5.377333  79.27433   6.037000         41.24467 112.47100 0.02966667
## 18     6.961667  56.19567   5.949000        103.21467  66.50367 0.05066667
## 19     6.002000  54.56567   5.682000        117.96167 295.55567 0.01900000
## 16     4.844333  88.94733   7.134667        147.61300 167.22333 0.04000000
## 15     8.683000  24.35800   5.483000         97.41000  42.50033 0.08633333
## 12     5.880333  50.33100   6.340000         87.68300 217.89733 0.05633333
## 13     6.352333  31.80733   4.821000        112.78467 162.83100 0.13366667
## 14     8.378333  56.07400   4.869000        134.27767 117.02067 0.15300000
## 11     7.007333  72.31300   7.801000        189.34433 158.28133 0.11166667
## 10    10.054000  14.15200   8.328000         77.32867 153.41767 0.49700000
## 7     10.023333  50.02633   5.885000        103.84233  89.09500 0.12400000
## 8      8.911667  13.26800   6.153000        157.18300  97.72367 0.36200000
## 9      4.387333  48.99033   6.938000        195.46300 183.69667 0.24433333
## 6     10.922333  55.69300   6.340000        269.82633 193.10967 0.00000000
## 5      9.642667  13.16167   7.526000        120.47200 222.76100 0.05633333
## 2      6.550333  73.66867   6.974000        103.52833 115.13767 0.08800000
## 3      8.576333  46.79700   6.865667        117.49100 122.82533 0.26600000
## 4      6.900667  32.12700   5.046000        156.71267 220.72133 0.14933333
## 1      7.982667  21.18933   8.736000        180.55900 252.88267 0.11200000
##          MnSOD     CuZnSOD  MalicAcid SuccinicAcid FumaricAcid Raffinose
## 20 0.027000000 0.014333333 0.24333333     3.136667   1.6633333  3.270000
## 17 0.006666667 0.024333333 0.24333333     3.136667   1.6633333  2.856667
## 18 0.021333333 0.028666667 0.09333333     4.550000   0.9533333  2.253333
## 19 0.001666667 0.017333333 0.11000000     5.600000   0.8700000  0.000000
## 16 0.002000000 0.037666667 0.14000000     2.840000   0.2333333  0.000000
## 15 0.000000000 0.086333333 0.07666667     2.246667   2.9700000  2.690000
## 12 0.000000000 0.056333333 0.02666667     4.200000   2.8266667  2.856667
## 13 0.000000000 0.133666667 0.08666667     2.553333   1.8533333  1.700000
## 14 0.000000000 0.153000000 0.07666667     2.320000   1.3200000  0.000000
## 11 0.000000000 0.111666667 0.28333333     2.126667   0.7966667  0.000000
## 10 0.528666667 0.003666667 0.13000000     4.403333   0.8600000  3.960000
## 7  0.073000000 0.101000000 0.12666667     5.233333   1.2300000  3.470000
## 8  0.107333333 0.254666667 0.09666667     4.400000   0.9900000  3.050000
## 9  0.071666667 0.173333333 0.03000000     4.660000   0.6300000  2.760000
## 6  0.121000000 0.000000000 0.12666667     3.926667   6.8133333  0.000000
## 5  0.011000000 0.045333333 0.52333333     4.086667   0.7733333  4.793333
## 2  0.492666667 0.000000000 1.36333333     2.883333   0.5766667  5.470000
## 3  0.048000000 0.218333333 0.57666667     3.643333   0.1700000  3.040000
## 4  0.052666667 0.137333333 0.33666667     4.463333   0.1100000  2.606667
## 1  0.131000000 0.033000000 0.16000000     4.273333   0.1233333  2.540000
##       Sucrose    Glucose  Fructose TotalSugars Scopoletin PolyPhenol
## 20  48.850000  35.940000  26.05667      114.12   25.98333   4.573333
## 17  49.203333  29.856667  26.78000      108.69   45.40000   6.160000
## 18  13.480000  67.113333  67.94000      150.79  120.80000   5.700000
## 19   5.236667  55.596667  62.40667      123.24  125.81333   3.906667
## 16   4.076667  18.140000  18.39667       40.62   66.64333   6.500000
## 15  48.506667  15.480000  13.69000       80.37   18.59000   4.240000
## 12  58.020000  30.233333  26.07333      117.18  124.89000   3.636667
## 13  10.400000  25.220000  31.62000       68.94   48.17000   4.256667
## 14   4.633333  17.093333  23.28333       45.01   81.79667   5.103333
## 11   3.233333   9.403333  13.41000       26.05   54.93667   3.820000
## 10  92.266667  82.263333  69.87333      248.37   64.25333   3.723333
## 7   62.050000  45.740000  42.72000      153.98   81.81333   3.400000
## 8   36.773333  82.080000  84.24667      206.15  123.90000   4.543333
## 9   19.490000  91.766667  99.79667      213.81  214.00000   2.980000
## 6    5.710000  42.230000  44.65333       92.59   98.10000   5.036667
## 5  112.100000  55.910000  54.19000      226.99   91.46000   4.190000
## 2   91.483333  87.296667  81.25000      265.50   92.10667   3.213333
## 3   25.523333 117.850000 125.74000      272.16   94.99667   3.643333
## 4   26.203333 102.346667 117.27333      248.43  193.95667   3.700000
## 1   28.196667  88.156667 111.09333      229.99  223.08000   6.533333
##     Ascorbic  Ascorbate  Guaiacol Tocopherol  Proteins PPDscores
## 20 0.5400000   1.320000 0.1366667  5.5766667  60.67333   0.00000
## 17 1.9400000  25.983333 1.3633333  0.7666667  66.89333  12.62667
## 18 1.4200000  27.896667 0.5233333  4.0400000  82.82333  50.45333
## 19 3.5666667  28.243333 1.2733333  2.0566667  65.74333  66.14667
## 16 2.6733333   7.066667 3.5800000  0.4233333  36.17333  77.47000
## 15 0.6433333  12.910000 0.1933333  0.3900000  39.32333   0.00000
## 12 0.7266667   3.743333 1.3433333  0.3166667  55.82333  41.08333
## 13 0.6300000  20.200000 1.5900000  0.2266667  69.17333  88.58000
## 14 1.8233333  16.033333 5.0700000  3.6166667  21.55000 102.00000
## 11 2.5833333 118.986667 6.5933333  0.2500000  13.07333 109.02667
## 10 0.2933333   2.820000 0.1400000  0.3900000  43.60333   0.00000
## 7  1.2466667  34.323333 1.0766667  2.1133333  55.39333  16.91333
## 8  1.0533333  19.436667 0.2366667  0.2566667 110.53333  55.06333
## 9  1.0366667   7.833333 1.8400000  0.3566667  57.46333  68.92000
## 6  1.3333333  73.730000 3.4466667  0.3500000  18.24333  87.61667
## 5  0.6333333  14.013333 0.2166667  0.2133333  37.39333   0.00000
## 2  2.3600000  31.050000 0.7733333  0.2500000  56.24333  39.29667
## 3  2.4300000  44.633333 0.8266667  0.2333333  52.60333  60.92333
## 4  2.6400000  26.456667 3.1133333  0.2500000  80.03667  85.37333
## 1  3.2033333   9.043333 1.1733333  2.5600000  27.32333  95.83000
```

### select column variables

```
feno<-dcast(means3[-c(4:31)],Days~Sample,fill=0)  ##select phenolics
flavo<-dcast(means3[-c(3,5:31)],Days~Sample,fill=0) ##select flavonoids
caro<-dcast(means3[-c(3:4,6:31)],Days~Sample,fill=0) ##carotenoids
anto<-dcast(means3[-c(3:5,7:31)],Days~Sample,fill=0) ##anthocyanins
hcn<-dcast(means3[-c(3:6,8:31)],Days~Sample,fill=0)  ##total cyanide
cn<-dcast(means3[-c(3:7,9:31)],Days~Sample,fill=0)##acetone cyanohydrin
lina<-dcast(means3[-c(3:8,10:31)],Days~Sample,fill=0) ##linamarin
linase<-dcast(means3[-c(3:9,11:31)],Days~Sample,fill=0)  ##linamarase
h2o2<-dcast(means3[-c(3:10,12:31)],Days~Sample,fill=0) ##hydrogen peroxide

cata<-dcast(means3[-c(3:11,13:31)],Days~Sample,fill=0) ##catalase
totsod<-dcast(means3[-c(3:12,14:31)],Days~Sample,fill=0)##total SOD
mnsod<-dcast(means3[-c(3:13,15:31)],Days~Sample,fill=0) ##MnSOD
cuznsod<-dcast(means3[-c(3:14,16:31)],Days~Sample,fill=0) ##CuZnSOD
malic<-dcast(means3[-c(3:15,17:31)],Days~Sample,fill=0) ##Malic acid
succ<-dcast(means3[-c(3:16,18:31)],Days~Sample,fill=0) ##Succinic acid
fum<-dcast(means3[-c(3:17,19:31)],Days~Sample,fill=0) ##Fumaric acid
raf<-dcast(means3[-c(3:18,20:31)],Days~Sample,fill=0) ##Raffinose
sucro<-dcast(means3[-c(3:19,21:31)],Days~Sample,fill=0) ##Sucrose
```

### PLOT FIGURE 1A.

```
windows()
par(mfrow=c(3,3))
plot_colors = c("blue","red","forestgreen","black")
plot(feno[-1]$BRA, type="o", col=plot_colors[1], ylim=c(0,max(feno[-1])), axes=FALSE, ann=FALSE,lwd=2)
axis(1, at=1:5, lab=c("0","3", "5", "8", "11"))
axis(2, las=1, at=200*0:max(feno[-1]))
lines(feno[-1]$BRA, type="o", col=plot_colors[1], lwd=2)
lines(feno[-1]$IAC, type="o", pch=22, lty=2,lwd=2, col=plot_colors[2])
lines(feno[-1]$ORI, type="o", pch=23, lty=3,lwd=2, col=plot_colors[3])
lines(feno[-1]$SAN, type="o", pch=23, lty=3, lwd=2, col=plot_colors[4])
title(xlab= "Storage days", col.lab="black")
title(ylab= "Phenolics", col.lab="black")

plot(flavo[-1]$BRA, type="o", col=plot_colors[1], ylim=c(0,max(flavo[-1])), axes=FALSE, ann=FALSE,lwd=2)
axis(1, at=1:5, lab=c("0","3", "5", "8", "11"))
axis(2, las=1, at=500*0:max(flavo[-1]))
lines(flavo[-1]$BRA, type="o", col=plot_colors[1], lwd=2)
lines(flavo[-1]$IAC, type="o", pch=22, lty=2,lwd=2, col=plot_colors[2])
lines(flavo[-1]$ORI, type="o", pch=23, lty=3,lwd=2, col=plot_colors[3])
lines(flavo[-1]$SAN, type="o", pch=23, lty=3, lwd=2, col=plot_colors[4])
title(xlab= "Storage days", col.lab="black")
title(ylab= "Flavonoids", col.lab="black")


plot(caro[-1]$BRA, type="o", col=plot_colors[1], ylim=c(0,max(caro[-1])), axes=FALSE, ann=FALSE,lwd=2)
axis(1, at=1:5, lab=c("0","3", "5", "8", "11"))
axis(2, las=1, at=2*0:max(caro[-1]))
lines(caro[-1]$BRA, type="o", col=plot_colors[1], lwd=2)
lines(caro[-1]$IAC, type="o", pch=22, lty=2,lwd=2, col=plot_colors[2])
lines(caro[-1]$ORI, type="o", pch=23, lty=3,lwd=2, col=plot_colors[3])
lines(caro[-1]$SAN, type="o", pch=23, lty=3, lwd=2, col=plot_colors[4])
title(xlab= "Storage days", col.lab="black")
title(ylab= "Carotenoids", col.lab="black")

  
plot(anto[-1]$BRA, type="o", col=plot_colors[1], ylim=c(0,max(anto[-1])), axes=FALSE, ann=FALSE,lwd=2)
axis(1, at=1:5, lab=c("0","3", "5", "8", "11"))
axis(2, las=1, at=10*0:max(anto[-1]))
lines(anto[-1]$BRA, type="o", col=plot_colors[1], lwd=2)
lines(anto[-1]$IAC, type="o", pch=22, lty=2,lwd=2, col=plot_colors[2])
lines(anto[-1]$ORI, type="o", pch=23, lty=3,lwd=2, col=plot_colors[3])
lines(anto[-1]$SAN, type="o", pch=23, lty=3, lwd=2, col=plot_colors[4])
title(xlab= "Storage days", col.lab="black")
title(ylab= "Anthocyanins", col.lab="black")
  
plot(hcn[-1]$BRA, type="o", col=plot_colors[1], ylim=c(0,max(hcn[-1])), axes=FALSE, ann=FALSE,lwd=2)
axis(1, at=1:5, lab=c("0","3", "5", "8", "11"))
axis(2, las=1, at=20*0:max(hcn[-1]))
lines(hcn[-1]$BRA, type="o", col=plot_colors[1], lwd=2)
lines(hcn[-1]$IAC, type="o", pch=22, lty=2,lwd=2, col=plot_colors[2])
lines(hcn[-1]$ORI, type="o", pch=23, lty=3,lwd=2, col=plot_colors[3])
lines(hcn[-1]$SAN, type="o", pch=23, lty=3, lwd=2, col=plot_colors[4])
title(xlab= "Storage days", col.lab="black")
title(ylab= "Total cyanide", col.lab="black")

  
plot(cn[-1]$BRA, type="o", col=plot_colors[1], ylim=c(0,max(cn[-1])), axes=FALSE, ann=FALSE,lwd=2)
axis(1, at=1:5, lab=c("0","3", "5", "8", "11"))
axis(2, las=1, at=3*0:max(cn[-1]))
lines(cn[-1]$BRA, type="o", col=plot_colors[1], lwd=2)
lines(cn[-1]$IAC, type="o", pch=22, lty=2,lwd=2, col=plot_colors[2])
lines(cn[-1]$ORI, type="o", pch=23, lty=3,lwd=2, col=plot_colors[3])
lines(cn[-1]$SAN, type="o", pch=23, lty=3, lwd=2, col=plot_colors[4])
title(xlab= "Storage days", col.lab="black")
title(ylab= "Acetone cyanohydrin", col.lab="black")

  
plot(h2o2[-1]$BRA, type="o", col=plot_colors[1], ylim=c(0,max(h2o2[-1])), axes=FALSE, ann=FALSE,lwd=2)
axis(1, at=1:5, lab=c("0","3", "5", "8", "11"))
axis(2, las=1, at=100*0:max(h2o2[-1]))
lines(h2o2[-1]$BRA, type="o", col=plot_colors[1], lwd=2)
lines(h2o2[-1]$IAC, type="o", pch=22, lty=2,lwd=2, col=plot_colors[2])
lines(h2o2[-1]$ORI, type="o", pch=23, lty=3,lwd=2, col=plot_colors[3])
lines(h2o2[-1]$SAN, type="o", pch=23, lty=3, lwd=2, col=plot_colors[4])
title(xlab= "Storage days", col.lab="black")
title(ylab= "Hydrogen peroxide", col.lab="black")

plot(lina[-1]$BRA, type="o", col=plot_colors[1], ylim=c(0,max(lina[-1])), axes=FALSE, ann=FALSE,lwd=2)
axis(1, at=1:5, lab=c("0","3", "5", "8", "11"))
axis(2, las=1, at=20*0:max(lina[-1]))
lines(lina[-1]$BRA, type="o", col=plot_colors[1], lwd=2)
lines(lina[-1]$IAC, type="o", pch=22, lty=2,lwd=2, col=plot_colors[2])
lines(lina[-1]$ORI, type="o", pch=23, lty=3,lwd=2, col=plot_colors[3])
lines(lina[-1]$SAN, type="o", pch=23, lty=3, lwd=2, col=plot_colors[4])
title(xlab= "Storage days", col.lab="black")
title(ylab= "Linamarin", col.lab="black")

plot(linase[-1]$BRA, type="o", col=plot_colors[1], ylim=c(0,max(linase[-1])), axes=FALSE, ann=FALSE,lwd=2)
axis(1, at=1:5, lab=c("0","3", "5", "8", "11"))
axis(2, las=1, at=2*0:max(linase[-1]))
lines(linase[-1]$BRA, type="o", col=plot_colors[1], lwd=2)
lines(linase[-1]$IAC, type="o", pch=22, lty=2,lwd=2, col=plot_colors[2])
lines(linase[-1]$ORI, type="o", pch=23, lty=3,lwd=2, col=plot_colors[3])
lines(linase[-1]$SAN, type="o", pch=23, lty=3, lwd=2, col=plot_colors[4])
title(xlab= "Storage days", col.lab="black")
title(ylab= "Linamarase", col.lab="black")
```

### PLOT FIGURE 1B

```
windows()
par(bg="cornsilk2")  ## background color
par(mfrow=c(3,3))
plot_colors = c("blue","red","forestgreen","black")
plot(cata[-1]$BRA, type="o", col=plot_colors[1], ylim=c(0,max(cata[-1])), axes=FALSE, ann=FALSE,lwd=2)
axis(1, at=1:5, lab=c("0","3", "5", "8", "11"))
axis(2, las=1, at=50*0:max(cata[-1]))
lines(cata[-1]$BRA, type="o", col=plot_colors[1], lwd=2)
lines(cata[-1]$IAC, type="o", pch=22, lty=2,lwd=2, col=plot_colors[2])
lines(cata[-1]$ORI, type="o", pch=23, lty=3,lwd=2, col=plot_colors[3])
lines(cata[-1]$SAN, type="o", pch=23, lty=3, lwd=2, col=plot_colors[4])
title(xlab= "Storage days", col.lab="black")
title(ylab= "Catalase", col.lab="black")

plot(totsod[-1]$BRA, type="o", col=plot_colors[1], ylim=c(0,max(totsod[-1])), axes=FALSE, ann=FALSE,lwd=2)
axis(1, at=1:5, lab=c("0","3", "5", "8", "11"))
axis(2, las=1)
lines(totsod[-1]$BRA, type="o", col=plot_colors[1], lwd=2)
lines(totsod[-1]$IAC, type="o", pch=22, lty=2,lwd=2, col=plot_colors[2])
lines(totsod[-1]$ORI, type="o", pch=23, lty=3,lwd=2, col=plot_colors[3])
lines(totsod[-1]$SAN, type="o", pch=23, lty=3, lwd=2, col=plot_colors[4])
title(xlab= "Storage days", col.lab="black")
title(ylab= "Total SOD", col.lab="black")


plot(mnsod[-1]$BRA, type="o", col=plot_colors[1], ylim=c(0,max(mnsod[-1])), axes=FALSE, ann=FALSE,lwd=2)
axis(1, at=1:5, lab=c("0","3", "5", "8", "11"))
axis(2, las=1)
lines(mnsod[-1]$BRA, type="o", col=plot_colors[1], lwd=2)
lines(mnsod[-1]$IAC, type="o", pch=22, lty=2,lwd=2, col=plot_colors[2])
lines(mnsod[-1]$ORI, type="o", pch=23, lty=3,lwd=2, col=plot_colors[3])
lines(mnsod[-1]$SAN, type="o", pch=23, lty=3, lwd=2, col=plot_colors[4])
title(xlab= "Storage days", col.lab="black")
title(ylab= "MnSOD", col.lab="black")


plot(cuznsod[-1]$BRA, type="o", col=plot_colors[1], ylim=c(0,max(cuznsod[-1])), axes=FALSE, ann=FALSE,lwd=2)
axis(1, at=1:5, lab=c("0","3", "5", "8", "11"))
axis(2, las=1)
lines(cuznsod[-1]$BRA, type="o", col=plot_colors[1], lwd=2)
lines(cuznsod[-1]$IAC, type="o", pch=22, lty=2,lwd=2, col=plot_colors[2])
lines(cuznsod[-1]$ORI, type="o", pch=23, lty=3,lwd=2, col=plot_colors[3])
lines(cuznsod[-1]$SAN, type="o", pch=23, lty=3, lwd=2, col=plot_colors[4])
title(xlab= "Storage days", col.lab="black")
title(ylab= "CuZnSOD", col.lab="black")

plot(malic[-1]$BRA, type="o", col=plot_colors[1], ylim=c(0,max(malic[-1])), axes=FALSE, ann=FALSE,lwd=2)
axis(1, at=1:5, lab=c("0","3", "5", "8", "11"))
axis(2, las=1)
lines(malic[-1]$BRA, type="o", col=plot_colors[1], lwd=2)
lines(malic[-1]$IAC, type="o", pch=22, lty=2,lwd=2, col=plot_colors[2])
lines(malic[-1]$ORI, type="o", pch=23, lty=3,lwd=2, col=plot_colors[3])
lines(malic[-1]$SAN, type="o", pch=23, lty=3, lwd=2, col=plot_colors[4])
title(xlab= "Storage days", col.lab="black")
title(ylab= "Malic acid", col.lab="black")


plot(succ[-1]$BRA, type="o", col=plot_colors[1], ylim=c(2,max(succ[-1])), axes=FALSE, ann=FALSE,lwd=2)
axis(1, at=1:5, lab=c("0","3", "5", "8", "11"))
axis(2, las=1,at=1*0:max(succ[-1]))
lines(succ[-1]$BRA, type="o", col=plot_colors[1], lwd=2)
lines(succ[-1]$IAC, type="o", pch=22, lty=2,lwd=2, col=plot_colors[2])
lines(succ[-1]$ORI, type="o", pch=23, lty=3,lwd=2, col=plot_colors[3])
lines(succ[-1]$SAN, type="o", pch=23, lty=3, lwd=2, col=plot_colors[4])
title(xlab= "Storage days", col.lab="black")
title(ylab= "Succinic Acid", col.lab="black")


plot(fum[-1]$BRA, type="o", col=plot_colors[1], ylim=c(0,max(fum[-1])), axes=FALSE, ann=FALSE,lwd=2)
axis(1, at=1:5, lab=c("0","3", "5", "8", "11"))
axis(2, las=1,at=1*0:max(fum[-1]))
lines(fum[-1]$BRA, type="o", col=plot_colors[1], lwd=2)
lines(fum[-1]$IAC, type="o", pch=22, lty=2,lwd=2, col=plot_colors[2])
lines(fum[-1]$ORI, type="o", pch=23, lty=3,lwd=2, col=plot_colors[3])
lines(fum[-1]$SAN, type="o", pch=23, lty=3, lwd=2, col=plot_colors[4])
title(xlab= "Storage days", col.lab="black")
title(ylab= "Fumaric Acid", col.lab="black")

plot(raf[-1]$BRA, type="o", col=plot_colors[1], ylim=c(0,max(raf[-1])), axes=FALSE, ann=FALSE,lwd=2)
axis(1, at=1:5, lab=c("0","3", "5", "8", "11"))
axis(2, las=1, at=1*0:max(raf[-1]))
lines(raf[-1]$BRA, type="o", col=plot_colors[1], lwd=2)
lines(raf[-1]$IAC, type="o", pch=22, lty=2,lwd=2, col=plot_colors[2])
lines(raf[-1]$ORI, type="o", pch=23, lty=3,lwd=2, col=plot_colors[3])
lines(raf[-1]$SAN, type="o", pch=23, lty=3, lwd=2, col=plot_colors[4])
title(xlab= "Storage days", col.lab="black")
title(ylab= "Raffinose", col.lab="black")

plot(sucro[-1]$BRA, type="o", col=plot_colors[1], ylim=c(0,max(sucro[-1])), axes=FALSE, ann=FALSE,lwd=2)
axis(1, at=1:5, lab=c("0","3", "5", "8", "11"))
axis(2, las=1, at=20*0:max(sucro[-1]))
lines(sucro[-1]$BRA, type="o", col=plot_colors[1], lwd=2)
lines(sucro[-1]$IAC, type="o", pch=22, lty=2,lwd=2, col=plot_colors[2])
lines(sucro[-1]$ORI, type="o", pch=23, lty=3,lwd=2, col=plot_colors[3])
lines(sucro[-1]$SAN, type="o", pch=23, lty=3, lwd=2, col=plot_colors[4])
title(xlab= "Storage days", col.lab="black")
title(ylab= "Sucrose", col.lab="black")
```

### FIGURE 2A-F

```
library(rattle) # GUI for building trees and fancy tree plot
library(rpart) # Popular decision tree algorithm
library(rpart.plot) # Enhanced tree plots
library(party) # Alternative decision tree algorithm
library(partykit) # Convert rpart object to BinaryTree
library(RWeka) # Weka decision tree J48.
library(C50) # Original C5.0 implementation.

##CALL DATA
setwd("C:/Users/Virgílio/Desktop/PASTAS DO DESKTOP/letter to ACS")

secMetabo<-read.csv("secMetabo.csv", header=T)  ##secondary metabolites
Cyanogenic<-read.csv("Cyanogenic.csv", header=T) ##cyanogenic glucosides
Enzymes<-read.csv("Enzymes.csv", header=T)  ##enzymes
SugarAcids<-read.csv("SugarAcids.csv", header=T)  ##Sugars and organic acids
ROS<-read.csv("ROS.csv", header=T)  ##scavenging reactive oxygen species
alldata<-read.csv("TabelaFinalTese2014.csv", header=T) ##all data sets combined
```

### Prepare the data and build decision trees for each data set

```
###prepare the data for secMetabo (A)
set.seed(12345)
ds <- secMetabo
target <- "PPDscores"
id <- c("Sample", "Days")
ignore <- id
nobs <- nrow(ds)
form <- formula(paste(target, "~ ."))
train <- sample(nobs, 0.70 * nobs)
(vars <- setdiff(names(ds), ignore))
```

```
## [1] "Phenolics"    "Flavonoids"   "Carotenoids"  "Anthocyanins"
## [5] "Scopoletin"   "PPDscores"
```

```
inputs <- setdiff(vars, target)
(nobs <- nrow(ds))
```

```
## [1] 60
```

```
(numerics <- intersect(inputs, names(ds)[which(sapply(ds[vars], is.numeric))]))
```

```
## [1] "Phenolics"    "Flavonoids"   "Carotenoids"  "Anthocyanins"
```

```
(categorics <- intersect(inputs, names(ds)[which(sapply(ds[vars], is.factor))]))
```

```
## character(0)
```

```
(form <- formula(paste(target, "~ .")))
```

```
## PPDscores ~ .
```

```
length(train <- sample(nobs, 0.7*nobs))
```

```
## [1] 42
```

```
length(test <- setdiff(seq_len(nobs), train))
```

```
## [1] 18
```

```
actual <- ds[test, target]

model <- rpart(formula=form, data=ds[train, vars])  ##metabo
summary(model)
```

```
## Call:
## rpart(formula = form, data = ds[train, vars])
##   n= 42 
## 
##           CP nsplit rel error    xerror      xstd
## 1 0.34021722      0 1.0000000 1.0306126 0.1864732
## 2 0.19650761      1 0.6597828 0.6922332 0.1558201
## 3 0.09293521      2 0.4632752 0.5441033 0.1139205
## 4 0.01000000      3 0.3703400 0.6304867 0.1381380
## 
## Variable importance
##    Phenolics   Flavonoids   Scopoletin  Carotenoids Anthocyanins 
##           42           23           17           14            3 
## 
## Node number 1: 42 observations,    complexity param=0.3402172
##   mean=51.42571, MSE=2119.986 
##   left son=2 (9 obs) right son=3 (33 obs)
##   Primary splits:
##       Phenolics    < 82.36    to the left,  improve=0.34021720, (0 missing)
##       Flavonoids   < 910      to the left,  improve=0.29419860, (0 missing)
##       Scopoletin   < 99.555   to the left,  improve=0.21119000, (0 missing)
##       Anthocyanins < 16.2815  to the left,  improve=0.03926710, (0 missing)
##       Carotenoids  < 3.281    to the left,  improve=0.02823829, (0 missing)
##   Surrogate splits:
##       Flavonoids  < 744.167  to the left,  agree=0.976, adj=0.889, (0 split)
##       Scopoletin  < 37.145   to the left,  agree=0.905, adj=0.556, (0 split)
##       Carotenoids < 2.257    to the left,  agree=0.881, adj=0.444, (0 split)
## 
## Node number 2: 9 observations
##   mean=0, MSE=0 
## 
## Node number 3: 33 observations,    complexity param=0.1965076
##   mean=65.45091, MSE=1780.202 
##   left son=6 (12 obs) right son=7 (21 obs)
##   Primary splits:
##       Phenolics    < 215.505  to the right, improve=0.29783680, (0 missing)
##       Carotenoids  < 3.97     to the right, improve=0.10768050, (0 missing)
##       Flavonoids   < 1355     to the right, improve=0.09484824, (0 missing)
##       Scopoletin   < 98.1     to the left,  improve=0.08863321, (0 missing)
##       Anthocyanins < 6.9305   to the left,  improve=0.02635477, (0 missing)
##   Surrogate splits:
##       Flavonoids   < 2039.166 to the right, agree=0.697, adj=0.167, (0 split)
##       Carotenoids  < 4.838    to the left,  agree=0.697, adj=0.167, (0 split)
##       Anthocyanins < 1.9205   to the left,  agree=0.697, adj=0.167, (0 split)
##       Scopoletin   < 51.175   to the left,  agree=0.697, adj=0.167, (0 split)
## 
## Node number 6: 12 observations
##   mean=34.99, MSE=615.4478 
## 
## Node number 7: 21 observations,    complexity param=0.09293521
##   mean=82.85714, MSE=1612.589 
##   left son=14 (7 obs) right son=15 (14 obs)
##   Primary splits:
##       Phenolics    < 123.995  to the left,  improve=0.24435400, (0 missing)
##       Carotenoids  < 4.942    to the right, improve=0.18509840, (0 missing)
##       Flavonoids   < 1359.166 to the right, improve=0.15249280, (0 missing)
##       Anthocyanins < 10.019   to the right, improve=0.03432596, (0 missing)
##       Scopoletin   < 98.1     to the left,  improve=0.02424582, (0 missing)
##   Surrogate splits:
##       Carotenoids  < 5.5905   to the right, agree=0.762, adj=0.286, (0 split)
##       Scopoletin   < 90.265   to the left,  agree=0.762, adj=0.286, (0 split)
##       Flavonoids   < 1571.666 to the right, agree=0.714, adj=0.143, (0 split)
##       Anthocyanins < 4.9265   to the left,  agree=0.714, adj=0.143, (0 split)
## 
## Node number 14: 7 observations
##   mean=54.78429, MSE=874.9343 
## 
## Node number 15: 14 observations
##   mean=96.89357, MSE=1390.353
```

```
prp(model, type=2, extra=101, nn=TRUE, fallen.leaves=TRUE,
faclen=0, varlen=0, shadow.col="orange", branch.lty=2,main="(A):Decision Regression Tree using Secondary Metabolites",cex.main=0.7)
```

```
###data for cyanogenics (B)
set.seed(12345)
ds1 <- Cyanogenic
target <- "PPDscores"
id <- c("Sample", "Days")
ignore <- id
nobs <- nrow(ds1)
form <- formula(paste(target, "~ ."))
train <- sample(nobs, 0.70 * nobs)
(vars <- setdiff(names(ds1), ignore))
```

```
## [1] "Totalcyanide" "AcetoneCyano" "Linamarin"    "Linamarase"  
## [5] "PPDscores"
```

```
inputs <- setdiff(vars, target)
(nobs <- nrow(ds1))
```

```
## [1] 60
```

```
(numerics <- intersect(inputs, names(ds1)[which(sapply(ds1[vars], is.numeric))]))
```

```
## [1] "Totalcyanide" "AcetoneCyano" "Linamarin"
```

```
(categorics <- intersect(inputs, names(ds1)[which(sapply(ds1[vars], is.factor))]))
```

```
## character(0)
```

```
(form <- formula(paste(target, "~ .")))
```

```
## PPDscores ~ .
```

```
length(train <- sample(nobs, 0.7*nobs))
```

```
## [1] 42
```

```
length(test <- setdiff(seq_len(nobs), train))
```

```
## [1] 18
```

```
actual <- ds1[test, target]

model1 <- rpart(formula=form, data=ds1[train, vars]) ##cyanogenics
summary(model1)
```

```
## Call:
## rpart(formula = form, data = ds1[train, vars])
##   n= 42 
## 
##          CP nsplit rel error   xerror      xstd
## 1 0.1960632      0 1.0000000 1.030613 0.1864732
## 2 0.0100000      2 0.6078736 1.236546 0.2447902
## 
## Variable importance
## AcetoneCyano    Linamarin Totalcyanide   Linamarase 
##           50           20           17           13 
## 
## Node number 1: 42 observations,    complexity param=0.1960632
##   mean=51.42571, MSE=2119.986 
##   left son=2 (14 obs) right son=3 (28 obs)
##   Primary splits:
##       AcetoneCyano < 8.523   to the right, improve=0.16427170, (0 missing)
##       Linamarin    < 55.914  to the left,  improve=0.13145570, (0 missing)
##       Totalcyanide < 62.632  to the left,  improve=0.12715540, (0 missing)
##       Linamarase   < 6.263   to the left,  improve=0.05326872, (0 missing)
##   Surrogate splits:
##       Totalcyanide < 26.2775 to the left,  agree=0.786, adj=0.357, (0 split)
##       Linamarin    < 17.2975 to the left,  agree=0.786, adj=0.357, (0 split)
## 
## Node number 2: 14 observations
##   mean=25.03429, MSE=1309.526 
## 
## Node number 3: 28 observations,    complexity param=0.1960632
##   mean=64.62143, MSE=2002.835 
##   left son=6 (19 obs) right son=7 (9 obs)
##   Primary splits:
##       AcetoneCyano < 6.9235  to the left,  improve=0.36177370, (0 missing)
##       Linamarase   < 7.148   to the left,  improve=0.16848290, (0 missing)
##       Linamarin    < 49.059  to the right, improve=0.14161540, (0 missing)
##       Totalcyanide < 53.766  to the right, improve=0.09665042, (0 missing)
##   Surrogate splits:
##       Linamarin    < 47.208  to the right, agree=0.821, adj=0.444, (0 split)
##       Linamarase   < 7.226   to the left,  agree=0.821, adj=0.444, (0 split)
##       Totalcyanide < 32.333  to the right, agree=0.786, adj=0.333, (0 split)
## 
## Node number 6: 19 observations
##   mean=46.09526, MSE=1167.04 
## 
## Node number 7: 9 observations
##   mean=103.7322, MSE=1513.064
```

```
printcp(model1)  ##see if is a regression model
```

```
## 
## Regression tree:
## rpart(formula = form, data = ds1[train, vars])
## 
## Variables actually used in tree construction:
## [1] AcetoneCyano
## 
## Root node error: 89039/42 = 2120
## 
## n= 42 
## 
##        CP nsplit rel error xerror    xstd
## 1 0.19606      0   1.00000 1.0306 0.18647
## 2 0.01000      2   0.60787 1.2365 0.24479
```

```
prp(model1, type=2, extra=101, nn=TRUE, fallen.leaves=TRUE,
faclen=0, varlen=0, shadow.col="orange", branch.lty=2,main="(B):Decision Regression Tree using Cyanogenic Glucosides",cex.main=0.7)
```

```
###data for enzymes (C)
set.seed(12345)
ds2 <- Enzymes
target <- "PPDscores"
id <- c("Sample", "Days")
ignore <- id
nobs <- nrow(ds2)
form <- formula(paste(target, "~ ."))
train <- sample(nobs, 0.70 * nobs)
(vars <- setdiff(names(ds2), ignore))
```

```
## [1] "PolyPhenol" "Ascorbic"   "Ascorbate"  "Guaiacol"   "Tocopherol"
## [6] "Proteins"   "PPDscores"
```

```
inputs <- setdiff(vars, target)
(nobs <- nrow(ds2))
```

```
## [1] 60
```

```
(numerics <- intersect(inputs, names(ds2)[which(sapply(ds2[vars], is.numeric))]))
```

```
## [1] "PolyPhenol" "Ascorbic"   "Ascorbate"  "Guaiacol"   "Tocopherol"
```

```
(categorics <- intersect(inputs, names(ds2)[which(sapply(ds2[vars], is.factor))]))
```

```
## character(0)
```

```
(form <- formula(paste(target, "~ .")))
```

```
## PPDscores ~ .
```

```
length(train <- sample(nobs, 0.7*nobs))
```

```
## [1] 42
```

```
length(test <- setdiff(seq_len(nobs), train))
```

```
## [1] 18
```

```
actual <- ds2[test, target]

model2 <- rpart(formula=form, data=ds2[train, vars])  ##enzymes
summary(model2)
```

```
## Call:
## rpart(formula = form, data = ds2[train, vars])
##   n= 42 
## 
##           CP nsplit rel error    xerror      xstd
## 1 0.38621897      0 1.0000000 1.0306126 0.1864732
## 2 0.17368229      1 0.6137810 0.9861663 0.1672450
## 3 0.06747828      2 0.4400987 0.7490577 0.1516850
## 4 0.01000000      3 0.3726205 0.7316289 0.1460642
## 
## Variable importance
##   Guaiacol   Proteins PolyPhenol   Ascorbic  Ascorbate Tocopherol 
##         27         26         15         15         14          3 
## 
## Node number 1: 42 observations,    complexity param=0.386219
##   mean=51.42571, MSE=2119.986 
##   left son=2 (31 obs) right son=3 (11 obs)
##   Primary splits:
##       Proteins   < 36.19  to the right, improve=0.38621900, (0 missing)
##       Guaiacol   < 1.79   to the left,  improve=0.37436460, (0 missing)
##       Ascorbic   < 1.015  to the left,  improve=0.36556880, (0 missing)
##       PolyPhenol < 4.88   to the left,  improve=0.17527060, (0 missing)
##       Ascorbate  < 39.37  to the left,  improve=0.09081982, (0 missing)
##   Surrogate splits:
##       Guaiacol   < 3.25   to the left,  agree=0.905, adj=0.636, (0 split)
##       PolyPhenol < 4.88   to the left,  agree=0.857, adj=0.455, (0 split)
##       Ascorbic   < 2.545  to the left,  agree=0.833, adj=0.364, (0 split)
##       Ascorbate  < 58.265 to the left,  agree=0.833, adj=0.364, (0 split)
## 
## Node number 2: 31 observations,    complexity param=0.1736823
##   mean=34.38065, MSE=1303.341 
##   left son=4 (10 obs) right son=5 (21 obs)
##   Primary splits:
##       Guaiacol   < 0.375  to the left,  improve=0.3827525, (0 missing)
##       Ascorbic   < 1.015  to the left,  improve=0.2965542, (0 missing)
##       PolyPhenol < 3.715  to the right, improve=0.2545725, (0 missing)
##       Proteins   < 48.07  to the left,  improve=0.1884146, (0 missing)
##       Ascorbate  < 19.665 to the left,  improve=0.1312751, (0 missing)
##   Surrogate splits:
##       Ascorbic   < 0.985  to the left,  agree=0.871, adj=0.6, (0 split)
##       Proteins   < 48.07  to the left,  agree=0.839, adj=0.5, (0 split)
##       PolyPhenol < 3.73   to the right, agree=0.806, adj=0.4, (0 split)
##       Ascorbate  < 2.63   to the left,  agree=0.806, adj=0.4, (0 split)
##       Tocopherol < 4.685  to the right, agree=0.774, adj=0.3, (0 split)
## 
## Node number 3: 11 observations
##   mean=99.46182, MSE=1295.193 
## 
## Node number 4: 10 observations
##   mean=2.014, MSE=36.50576 
## 
## Node number 5: 21 observations,    complexity param=0.06747828
##   mean=49.79333, MSE=1170.188 
##   left son=10 (14 obs) right son=11 (7 obs)
##   Primary splits:
##       Guaiacol   < 1.36   to the left,  improve=0.24449580, (0 missing)
##       Tocopherol < 0.36   to the right, improve=0.17478930, (0 missing)
##       Proteins   < 56.87  to the left,  improve=0.12476490, (0 missing)
##       Ascorbate  < 28.51  to the right, improve=0.11100170, (0 missing)
##       Ascorbic   < 2.27   to the left,  improve=0.04312791, (0 missing)
##   Surrogate splits:
##       Ascorbate  < 23.095 to the right, agree=0.905, adj=0.714, (0 split)
##       PolyPhenol < 3.12   to the right, agree=0.810, adj=0.429, (0 split)
##       Ascorbic   < 1.15   to the right, agree=0.810, adj=0.429, (0 split)
##       Proteins   < 56.87  to the left,  agree=0.714, adj=0.143, (0 split)
## 
## Node number 10: 14 observations
##   mean=37.83286, MSE=827.5443 
## 
## Node number 11: 7 observations
##   mean=73.71429, MSE=997.157
```

```
prp(model2, type=2, extra=101, nn=TRUE, fallen.leaves=TRUE,
faclen=0, varlen=0, shadow.col="orange", branch.lty=2,main="(C):Decision Regression Tree using Enzymes",cex.main=0.7)
```

```
### data for Sugar and Acids (D)
set.seed(12345)
ds3 <- SugarAcids
target <- "PPDscores"
id <- c("Sample", "Days")
ignore <- id
nobs <- nrow(ds3)
form <- formula(paste(target, "~ ."))
train <- sample(nobs, 0.70 * nobs)
(vars <- setdiff(names(ds3), ignore))
```

```
## [1] "MalicAcid"    "SuccinicAcid" "FumaricAcid"  "Raffinose"   
## [5] "Sucrose"      "Glucose"      "Fructose"     "TotalSugars" 
## [9] "PPDscores"
```

```
inputs <- setdiff(vars, target)
(nobs <- nrow(ds3))
```

```
## [1] 60
```

```
(numerics <- intersect(inputs, names(ds3)[which(sapply(ds3[vars], is.numeric))]))
```

```
## [1] "MalicAcid"    "SuccinicAcid" "FumaricAcid"  "Raffinose"   
## [5] "Sucrose"      "Glucose"      "Fructose"
```

```
(categorics <- intersect(inputs, names(ds3)[which(sapply(ds3[vars], is.factor))]))
```

```
## character(0)
```

```
(form <- formula(paste(target, "~ .")))
```

```
## PPDscores ~ .
```

```
length(train <- sample(nobs, 0.7*nobs))
```

```
## [1] 42
```

```
length(test <- setdiff(seq_len(nobs), train))
```

```
## [1] 18
```

```
actual <- ds3[test, target]

model3 <- rpart(formula=form, data=ds3[train, vars])  ## sugar and acids
summary(model3)
```

```
## Call:
## rpart(formula = form, data = ds3[train, vars])
##   n= 42 
## 
##           CP nsplit rel error    xerror      xstd
## 1 0.52182910      0 1.0000000 1.0306126 0.1864732
## 2 0.04402899      1 0.4781709 0.5134705 0.1138624
## 3 0.01000000      2 0.4341419 0.7210448 0.1609335
## 
## Variable importance
##      Sucrose    Raffinose     Fructose  FumaricAcid      Glucose 
##           33           23           12           12            9 
##    MalicAcid SuccinicAcid  TotalSugars 
##            7            3            2 
## 
## Node number 1: 42 observations,    complexity param=0.5218291
##   mean=51.42571, MSE=2119.986 
##   left son=2 (19 obs) right son=3 (23 obs)
##   Primary splits:
##       Sucrose     < 33.845 to the right, improve=0.5218291, (0 missing)
##       Raffinose   < 2.765  to the right, improve=0.3942982, (0 missing)
##       FumaricAcid < 0.81   to the right, improve=0.2041970, (0 missing)
##       TotalSugars < 56.975 to the right, improve=0.1969301, (0 missing)
##       Fructose    < 92.71  to the left,  improve=0.1273802, (0 missing)
##   Surrogate splits:
##       Raffinose   < 3.075  to the right, agree=0.857, adj=0.684, (0 split)
##       FumaricAcid < 0.465  to the right, agree=0.714, adj=0.368, (0 split)
##       Fructose    < 92.71  to the left,  agree=0.690, adj=0.316, (0 split)
##       MalicAcid   < 0.085  to the right, agree=0.643, adj=0.211, (0 split)
##       Glucose     < 87.73  to the left,  agree=0.643, adj=0.211, (0 split)
## 
## Node number 2: 19 observations
##   mean=14.83105, MSE=483.5404 
## 
## Node number 3: 23 observations,    complexity param=0.04402899
##   mean=81.65609, MSE=1451.686 
##   left son=6 (16 obs) right son=7 (7 obs)
##   Primary splits:
##       SuccinicAcid < 2.895  to the right, improve=0.11741410, (0 missing)
##       Raffinose    < 2.58   to the right, improve=0.09735484, (0 missing)
##       Glucose      < 42.23  to the right, improve=0.08458517, (0 missing)
##       Fructose     < 44.655 to the right, improve=0.08458517, (0 missing)
##       Sucrose      < 5.19   to the right, improve=0.07201502, (0 missing)
##   Surrogate splits:
##       Glucose     < 18.53  to the right, agree=0.957, adj=0.857, (0 split)
##       Fructose    < 37.765 to the right, agree=0.957, adj=0.857, (0 split)
##       TotalSugars < 80.765 to the right, agree=0.957, adj=0.857, (0 split)
##       Sucrose     < 5.19   to the right, agree=0.913, adj=0.714, (0 split)
##       Raffinose   < 1.91   to the right, agree=0.826, adj=0.429, (0 split)
## 
## Node number 6: 16 observations
##   mean=73.02062, MSE=1217.297 
## 
## Node number 7: 7 observations
##   mean=101.3943, MSE=1427.387
```

```
prp(model3, type=2, extra=101, nn=TRUE, fallen.leaves=TRUE,
faclen=0, varlen=0, shadow.col="orange", branch.lty=2,main="(D):Decision Regression Tree using Sugars and Organic Acids",cex.main=0.7)
```

```
### Scavenging Reative oxygen species (E)
set.seed(12345)
ds4 <- ROS
target <- "PPDscores"
id <- c("Sample", "Days")
ignore <- id
nobs <- nrow(ds4)
form <- formula(paste(target, "~ ."))
train <- sample(nobs, 0.70 * nobs)
(vars <- setdiff(names(ds4), ignore))
```

```
## [1] "HydrogenPeroxide" "Catalase"         "TotalSOD"        
## [4] "MnSOD"            "CuZnSOD"          "PPDscores"
```

```
inputs <- setdiff(vars, target)
(nobs <- nrow(ds4))
```

```
## [1] 60
```

```
(numerics <- intersect(inputs, names(ds4)[which(sapply(ds4[vars], is.numeric))]))
```

```
## [1] "HydrogenPeroxide" "Catalase"         "TotalSOD"        
## [4] "MnSOD"
```

```
(categorics <- intersect(inputs, names(ds4)[which(sapply(ds4[vars], is.factor))]))
```

```
## character(0)
```

```
(form <- formula(paste(target, "~ .")))
```

```
## PPDscores ~ .
```

```
length(train <- sample(nobs, 0.7*nobs))
```

```
## [1] 42
```

```
length(test <- setdiff(seq_len(nobs), train))
```

```
## [1] 18
```

```
actual <- ds4[test, target]


model4 <- rpart(formula=form, data=ds4[train, vars])  ## SCAVENGING Reactive species
summary(model4)
```

```
## Call:
## rpart(formula = form, data = ds4[train, vars])
##   n= 42 
## 
##           CP nsplit rel error    xerror      xstd
## 1 0.42045949      0 1.0000000 1.0306126 0.1864732
## 2 0.04284409      1 0.5795405 0.6715438 0.1216672
## 3 0.01000000      2 0.5366964 0.7626752 0.1237643
## 
## Variable importance
## HydrogenPeroxide         Catalase          CuZnSOD            MnSOD 
##               57               21                9                7 
##         TotalSOD 
##                6 
## 
## Node number 1: 42 observations,    complexity param=0.4204595
##   mean=51.42571, MSE=2119.986 
##   left son=2 (25 obs) right son=3 (17 obs)
##   Primary splits:
##       HydrogenPeroxide < 127.6885 to the left,  improve=0.42045950, (0 missing)
##       Catalase         < 98.508   to the left,  improve=0.18286030, (0 missing)
##       CuZnSOD          < 0.086    to the left,  improve=0.14167750, (0 missing)
##       TotalSOD         < 0.1045   to the left,  improve=0.10560780, (0 missing)
##       MnSOD            < 0.0185   to the right, improve=0.05389475, (0 missing)
##   Surrogate splits:
##       Catalase < 121.57   to the left,  agree=0.738, adj=0.353, (0 split)
##       CuZnSOD  < 0.086    to the left,  agree=0.667, adj=0.176, (0 split)
##       TotalSOD < 0.081    to the left,  agree=0.643, adj=0.118, (0 split)
##       MnSOD    < 0.0745   to the left,  agree=0.643, adj=0.118, (0 split)
## 
## Node number 2: 25 observations,    complexity param=0.04284409
##   mean=26.806, MSE=941.7524 
##   left son=4 (9 obs) right son=5 (16 obs)
##   Primary splits:
##       HydrogenPeroxide < 99.92    to the left,  improve=0.16203040, (0 missing)
##       TotalSOD         < 0.0425   to the left,  improve=0.13758140, (0 missing)
##       CuZnSOD          < 0.0065   to the left,  improve=0.07977996, (0 missing)
##       Catalase         < 89.801   to the left,  improve=0.06416749, (0 missing)
##       MnSOD            < 0.0185   to the right, improve=0.02020611, (0 missing)
##   Surrogate splits:
##       Catalase < 52.384   to the left,  agree=0.84, adj=0.556, (0 split)
##       MnSOD    < 0.0025   to the left,  agree=0.68, adj=0.111, (0 split)
## 
## Node number 3: 17 observations
##   mean=87.63118, MSE=1650.478 
## 
## Node number 4: 9 observations
##   mean=10.33556, MSE=507.2742 
## 
## Node number 5: 16 observations
##   mean=36.07062, MSE=947.7207
```

```
prp(model4, type=2, extra=101, nn=TRUE, fallen.leaves=TRUE,
faclen=0, varlen=0, shadow.col="orange", branch.lty=2,main="(E):Decision Regression Tree using Reactive Oxygen Species",cex.main=0.7)
```

```
## all data set (F)
set.seed(12345)
ds5 <- alldata
target <- "PPDscores"
id <- c("Sample", "Days")
ignore <- id
nobs <- nrow(ds5)
form <- formula(paste(target, "~ ."))
train <- sample(nobs, 0.70 * nobs)
(vars <- setdiff(names(ds5), ignore))
```

```
##  [1] "Phenolics"        "Flavonoids"       "Carotenoids"     
##  [4] "Anthocyanins"     "Totalcyanide"     "AcetoneCyano"    
##  [7] "Linamarin"        "Linamarase"       "HydrogenPeroxide"
## [10] "Catalase"         "TotalSOD"         "MnSOD"           
## [13] "CuZnSOD"          "MalicAcid"        "SuccinicAcid"    
## [16] "FumaricAcid"      "Raffinose"        "Sucrose"         
## [19] "Glucose"          "Fructose"         "TotalSugars"     
## [22] "Scopoletin"       "PolyPhenol"       "Ascorbic"        
## [25] "Ascorbate"        "Guaiacol"         "Tocopherol"      
## [28] "Proteins"         "PPDscores"
```

```
inputs <- setdiff(vars, target)
(nobs <- nrow(ds5))
```

```
## [1] 60
```

```
(numerics <- intersect(inputs, names(ds5)[which(sapply(ds5[vars], is.numeric))]))
```

```
##  [1] "Phenolics"        "Flavonoids"       "Carotenoids"     
##  [4] "Anthocyanins"     "Totalcyanide"     "AcetoneCyano"    
##  [7] "Linamarin"        "Linamarase"       "HydrogenPeroxide"
## [10] "Catalase"         "TotalSOD"         "MnSOD"           
## [13] "CuZnSOD"          "MalicAcid"        "SuccinicAcid"    
## [16] "FumaricAcid"      "Raffinose"        "Sucrose"         
## [19] "Glucose"          "Fructose"         "TotalSugars"     
## [22] "Scopoletin"       "PolyPhenol"       "Ascorbic"        
## [25] "Ascorbate"        "Guaiacol"         "Tocopherol"
```

```
(categorics <- intersect(inputs, names(ds5)[which(sapply(ds5[vars], is.factor))]))
```

```
## character(0)
```

```
(form <- formula(paste(target, "~ .")))
```

```
## PPDscores ~ .
```

```
length(train <- sample(nobs, 0.7*nobs))
```

```
## [1] 42
```

```
length(test <- setdiff(seq_len(nobs), train))
```

```
## [1] 18
```

```
actual <- ds5[test, target]

model5 <- rpart(formula=form, data=ds5[train, vars])   ## all dataset
summary(model5)
```

```
## Call:
## rpart(formula = form, data = ds5[train, vars])
##   n= 42 
## 
##           CP nsplit rel error    xerror      xstd
## 1 0.52182910      0 1.0000000 1.0306126 0.1864732
## 2 0.08156605      1 0.4781709 0.5134705 0.1138624
## 3 0.01000000      2 0.3966049 0.7678115 0.1697925
## 
## Variable importance
##          Sucrose HydrogenPeroxide        Raffinose         Guaiacol 
##               22               15               15               14 
##         Ascorbic        Phenolics      Carotenoids     SuccinicAcid 
##               14               10                3                2 
##       Flavonoids         Proteins       Scopoletin 
##                2                2                2 
## 
## Node number 1: 42 observations,    complexity param=0.5218291
##   mean=51.42571, MSE=2119.986 
##   left son=2 (19 obs) right son=3 (23 obs)
##   Primary splits:
##       Sucrose          < 33.845   to the right, improve=0.5218291, (0 missing)
##       HydrogenPeroxide < 127.6885 to the left,  improve=0.4204595, (0 missing)
##       Raffinose        < 2.765    to the right, improve=0.3942982, (0 missing)
##       Proteins         < 36.19    to the right, improve=0.3862190, (0 missing)
##       Guaiacol         < 1.79     to the left,  improve=0.3743646, (0 missing)
##   Surrogate splits:
##       HydrogenPeroxide < 109.333  to the left,  agree=0.857, adj=0.684, (0 split)
##       Raffinose        < 3.075    to the right, agree=0.857, adj=0.684, (0 split)
##       Ascorbic         < 0.985    to the left,  agree=0.833, adj=0.632, (0 split)
##       Guaiacol         < 0.81     to the left,  agree=0.810, adj=0.579, (0 split)
##       Phenolics        < 82.36    to the left,  agree=0.762, adj=0.474, (0 split)
## 
## Node number 2: 19 observations
##   mean=14.83105, MSE=483.5404 
## 
## Node number 3: 23 observations,    complexity param=0.08156605
##   mean=81.65609, MSE=1451.686 
##   left son=6 (14 obs) right son=7 (9 obs)
##   Primary splits:
##       Carotenoids      < 4.653    to the right, improve=0.2175159, (0 missing)
##       Proteins         < 27.34    to the right, improve=0.2066228, (0 missing)
##       HydrogenPeroxide < 126.747  to the left,  improve=0.1635979, (0 missing)
##       Guaiacol         < 1.79     to the left,  improve=0.1539508, (0 missing)
##       MnSOD            < 0.003    to the right, improve=0.1379522, (0 missing)
##   Surrogate splits:
##       SuccinicAcid < 2.5      to the right, agree=0.826, adj=0.556, (0 split)
##       Flavonoids   < 1112.5   to the right, agree=0.783, adj=0.444, (0 split)
##       Scopoletin   < 63.06    to the right, agree=0.783, adj=0.444, (0 split)
##       Guaiacol     < 3.495    to the left,  agree=0.783, adj=0.444, (0 split)
##       Proteins     < 24.47    to the right, agree=0.783, adj=0.444, (0 split)
## 
## Node number 6: 14 observations
##   mean=67.40857, MSE=1066.812 
## 
## Node number 7: 9 observations
##   mean=103.8189, MSE=1243.425
```

```
prp(model5, type=2, extra=101, nn=TRUE, fallen.leaves=TRUE,
faclen=0, varlen=0, shadow.col="orange", branch.lty=2,main="(F):Decision Regression Tree using all dataset",cex.main=0.7)
```

### FIGURE 3 (RIGHT)

```
setwd("C:/Users/Virgílio/Desktop/PASTAS DO DESKTOP/ascorbato peroxidase")
load("newdata.RData")
newdata_melt <- melt(newdata, id = c("Sample", "Days"))
means<-dcast(newdata_melt, Sample + Days ~ variable, mean)  ## all means
sd<-dcast(newdata_melt, Sample + Days ~ variable, sd)   ##all standard deviations

ppd<-means[-c(3:6)]  ##call means ppd
ppd2=matrix(ppd$asinPPD,5,4)  ##transform to matrix
rownames(ppd2) = levels(ppd$Days)
colnames(ppd2) = levels(ppd$Sample)
ppd2
```

```
##            BRA      IAC       ORI      SAN
## Fresh  0.00000  0.00000   0.00000  0.00000
## Day3  39.29667 16.91333  41.08333 12.62667
## Day5  60.92333 55.06333  88.58000 50.45333
## Day8  85.37333 68.92000 102.00000 66.14667
## Day11 95.83000 87.61667 109.02667 77.47000
```

```
SD<-sd[-c(3:6)]  ##call standard deviation of tocopherol
SD2=matrix(SD$asinPPD,5,4)
rownames(SD2) = levels(SD$Days)
colnames(SD2) = levels(SD$Sample)
SD2
```

```
##            BRA      IAC      ORI      SAN
## Fresh  0.00000  0.00000  0.00000  0.00000
## Day3  26.12894 16.99559 31.48827 14.11173
## Day5  41.27605 30.65746 60.76923 27.26702
## Day8  46.85692 33.61286 49.77227 34.61916
## Day11 37.65194 31.26715 43.31654 35.35903
```

```
mydata<-ppd2[-1,]  ##delete first row
SD3<-SD2[-1,]

#### Function to plot "one end" error bar

superpose.eb <- function (x, y, ebl, ebu = ebl, length = 0.08, ...)
  arrows(x, y + ebu, x, y - ebl, angle = 90, code = 3,
         length = length, ...)

require(TeachingDemos)
fillcolours = c("aliceblue","antiquewhite4","aquamarine4","chartreuse4")

x.abscis <- barplot(
  mydata, beside=TRUE,
  col=fillcolours, # makes sense in the context of width and space parameters
  space=c(0.4,2), # spacing between bars in the same group, and then between groups
  ylim=c(0,250),
  xlab="Cassava cultivars",axis.lty=1, # enable tick marks on the X axis
  font.lab=2, # bold for axis labels
  ylab="PPD Scores (Arcsin PPD)")
superpose.eb(x.abscis, mydata, ebl=0, ebu=SD3)
legend(x=2, y=225, box.lty=0, legend=c(rownames(mydata)), fill=fillcolours)
```

### TABLE 1

```
setwd("C:/Users/Virgílio/Desktop/PASTAS DO DESKTOP/ascorbato peroxidase")
load("TabelaFinalTese2014.RData")  ##load data
TabelaFinalTese2014
```

```
##    Sample  Days Phenolics Flavonoids Carotenoids Anthocyanins Totalcyanide
## 1     SAN Fresh     41.38    577.500       1.435        1.837       60.096
## 2     SAN Fresh     44.21    562.500       5.312        8.182       55.297
## 3     SAN Fresh     42.14    595.833       4.954       11.355       55.206
## 4     ORI Fresh     61.97    739.167       1.343        5.845       36.194
## 5     ORI Fresh     54.97    760.833       2.894        4.008       32.219
## 6     ORI Fresh     75.09    817.500       4.838        5.177       30.710
## 7     IAC Fresh     71.57    385.833       4.306        8.516       24.632
## 8     IAC Fresh     65.22    380.833       4.190       15.196       24.221
## 9     IAC Fresh     66.42    427.500       4.537       15.864       23.764
## 10    BRA Fresh     78.74    495.833       1.597        9.518       22.850
## 11    BRA Fresh     66.04    490.833       1.493        7.681       22.850
## 12    BRA Fresh     55.60    540.833       1.458        1.837       22.713
## 13    SAN  Day3    299.81   1049.167       4.745        9.518       86.967
## 14    SAN  Day3    338.93    932.500       4.525        4.342       84.956
## 15    SAN  Day3    463.15    895.833       4.630       10.520       82.032
## 16    ORI  Day3    558.99   1460.833       4.641        9.518       58.679
## 17    ORI  Day3    545.60   1465.833       5.567       16.866       55.206
## 18    ORI  Day3    531.07    970.833       5.046       16.365       54.749
## 19    IAC  Day3    524.97   1514.167       5.046        1.169       59.227
## 20    IAC  Day3    539.43   1359.167       4.132        5.511       60.324
## 21    IAC  Day3    493.43   1247.500       3.171       10.854       60.598
## 22    BRA  Day3    538.62   2942.500       3.414        0.000       80.889
## 23    BRA  Day3    543.33   2410.833       4.757        0.668       80.249
## 24    BRA  Day3    499.12   1510.833       4.479       16.198       79.518
## 25    SAN  Day5     95.79   1407.500       7.951       16.365       66.265
## 26    SAN  Day5    167.93   1394.167       6.227       15.864       64.254
## 27    SAN  Day5    111.76   1422.500       5.949       16.532       58.953
## 28    ORI  Day5    143.84    859.167       4.977        5.177       39.942
## 29    ORI  Day5    171.64    735.833       5.382        6.179       39.576
## 30    ORI  Day5    225.03    749.167       4.201        5.177       34.961
## 31    IAC  Day5    141.82   1609.167       4.722        0.668       22.622
## 32    IAC  Day5    116.42   1667.500       4.630        0.000       22.165
## 33    IAC  Day5    162.96   1734.167       3.785        0.835       21.753
## 34    BRA  Day5    131.13   1555.833       4.919        8.015       56.668
## 35    BRA  Day5    118.43   1587.500       5.000        8.683       55.388
## 36    BRA  Day5    210.63   1607.500       5.093       10.186       54.063
## 37    SAN  Day8    114.09   1295.833       6.146       11.689       61.695
## 38    SAN  Day8    190.57   1340.833       6.215       20.373       60.781
## 39    SAN  Day8    165.91   1315.833       4.514       25.048       59.227
## 40    ORI  Day8    164.53   1052.500       3.391        2.672       66.265
## 41    ORI  Day8    225.72   1052.500       3.808        8.683       64.483
## 42    ORI  Day8    169.37   1059.167       3.785        6.179       62.609
## 43    IAC  Day8    209.43   1025.833       2.917        8.015       53.469
## 44    IAC  Day8    220.38   1025.833       4.676        6.680       53.469
## 45    IAC  Day8    164.28   1002.500       3.426        5.678       53.195
## 46    BRA  Day8    187.30   1172.500       5.208        9.852       39.165
## 47    BRA  Day8    224.09   1145.833       5.706       10.520       39.074
## 48    BRA  Day8    205.79   1192.500       4.931       15.029       38.845
## 49    SAN Day11     93.46   1189.167       7.708        4.175       93.228
## 50    SAN Day11     85.98   1262.500       5.741        8.015       93.685
## 51    SAN Day11    129.56   1240.833       8.356        7.181       94.462
## 52    ORI Day11    158.30   1350.833       0.984        8.850       79.061
## 53    ORI Day11     88.43   1367.500       4.236       10.520       79.472
## 54    ORI Day11    131.01   1377.500       4.178       12.858       79.427
## 55    IAC Day11    133.02   1334.167       4.630       22.877       68.687
## 56    IAC Day11    179.81   1404.167       5.440       21.375       66.265
## 57    IAC Day11    119.56   1422.500       4.190       22.210       64.894
## 58    BRA Day11    203.02   1512.500       4.595       43.083       29.705
## 59    BRA Day11    191.07   1415.833       4.965       41.580       29.020
## 60    BRA Day11    152.14   1407.500       5.127       44.085       28.791
##    AcetoneCyano Linamarin Linamarase HydrogenPeroxide Catalase TotalSOD
## 1         4.387    55.708      6.055           61.797   17.085    0.029
## 2         3.290    52.007      6.157           62.268   13.320    0.041
## 3         3.245    51.961      6.194           62.738   14.261    0.048
## 4         8.729    27.466      5.477           97.567   37.794    0.021
## 5         8.683    23.535      5.483           97.567   42.500    0.087
## 6         8.637    22.073      5.489           97.096   47.207    0.151
## 7        10.100    14.533      8.312           75.917  126.747    0.461
## 8        10.054    14.167      8.330           76.858  150.280    0.533
## 9        10.008    13.756      8.342           79.211  183.226    0.497
## 10        9.688    13.162      7.518          119.217  203.935    0.000
## 11        9.643    13.207      7.524          121.570  228.879    0.096
## 12        9.597    13.116      7.536          120.629  235.469    0.073
## 13        5.438    81.529      6.019           40.617  112.628    0.016
## 14        5.393    79.564      6.037           41.088  108.392    0.033
## 15        5.301    76.730      6.055           42.029  116.393    0.040
## 16        5.941    52.738      6.332           86.271  202.993    0.044
## 17        5.850    49.356      6.338           88.154  214.760    0.068
## 18        5.850    48.899      6.350           88.624  235.939    0.057
## 19       10.145    49.082      5.881          103.685   87.683    0.344
## 20       10.008    50.316      5.887          103.215   89.095    0.028
## 21        9.917    50.681      5.887          104.627   90.507    0.000
## 22        6.627    74.263      6.976          102.744  107.921    0.249
## 23        6.535    73.714      6.970          102.273  117.805    0.000
## 24        6.489    73.029      6.976          105.568  119.687    0.015
## 25        7.038    59.227      5.947          102.273   70.269    0.057
## 26        6.992    57.262      5.947          104.627   71.681    0.040
## 27        6.855    52.098      5.953          102.744   57.561    0.055
## 28        6.398    33.544      4.797          112.628  150.751    0.133
## 29        6.352    33.224      4.815          112.628  162.517    0.132
## 30        6.307    28.654      4.851          113.098  175.225    0.136
## 31        8.957    13.664      6.151          158.281   89.095    0.356
## 32        8.912    13.253      6.151          157.340   95.684    0.395
## 33        8.866    12.887      6.157          155.928  108.392    0.335
## 34        8.683    47.985      6.621          115.452  119.217    0.258
## 35        8.546    46.843      6.687          117.334  132.395    0.275
## 36        8.500    45.563      7.289          119.687  116.864    0.265
## 37        6.078    55.617      5.676          119.217  299.007    0.020
## 38        5.987    54.794      5.682          117.334  286.770    0.018
## 39        5.941    53.286      5.688          117.334  300.890    0.019
## 40        8.455    57.810      4.839          133.807  101.332    0.155
## 41        8.363    56.120      4.869          133.807  123.453    0.155
## 42        8.317    54.292      4.899          135.219  126.277    0.149
## 43        4.433    49.036      6.934          194.992  192.639    0.285
## 44        4.387    49.082      6.940          195.463  176.166    0.359
## 45        4.342    48.853      6.940          195.934  182.285    0.089
## 46        6.946    32.218      5.044          154.987  210.053    0.405
## 47        6.901    32.173      5.044          157.811  204.405    0.043
## 48        6.855    31.990      5.050          157.340  247.706    0.000
## 49        4.890    88.338      7.108          147.456  160.634    0.045
## 50        4.844    88.841      7.133          148.868  169.106    0.054
## 51        4.799    89.663      7.163          146.515  171.930    0.021
## 52        7.266    71.795      7.789          188.874  162.517    0.113
## 53        6.901    72.572      7.807          189.815  154.987    0.126
## 54        6.855    72.572      7.807          189.344  157.340    0.096
## 55       11.014    57.673      6.338          270.297  153.104    0.000
## 56       10.922    55.343      6.338          269.356  214.760    0.000
## 57       10.831    54.063      6.344          269.826  211.465    0.000
## 58        7.998    21.707      8.728          178.990  245.352    0.177
## 59        7.998    21.022      8.740          180.873  258.060    0.126
## 60        7.952    20.839      8.740          181.814  255.236    0.033
##    MnSOD CuZnSOD MalicAcid SuccinicAcid FumaricAcid Raffinose Sucrose
## 1  0.027   0.002      0.28         3.18        1.66      3.14   46.62
## 2  0.000   0.041      0.31         3.11        1.78      3.40   51.08
## 3  0.054   0.000      0.14         3.12        1.55      3.27   48.85
## 4  0.000   0.021      0.06         2.12        2.74      2.56   45.60
## 5  0.000   0.087      0.06         2.40        3.06      2.82   51.41
## 6  0.000   0.151      0.11         2.22        3.11      2.69   48.51
## 7  0.528   0.000      0.13         4.34        0.81      3.83   89.98
## 8  0.522   0.011      0.14         4.48        0.84      4.09   94.55
## 9  0.536   0.000      0.12         4.39        0.93      3.96   92.27
## 10 0.000   0.000      0.48         3.87        0.82      4.65  108.00
## 11 0.033   0.063      0.52         4.20        0.90      4.94  116.20
## 12 0.000   0.073      0.57         4.19        0.60      4.79  112.10
## 13 0.020   0.000      0.28         3.18        1.66      2.77   47.49
## 14 0.000   0.033      0.31         3.11        1.78      2.94   50.92
## 15 0.000   0.040      0.14         3.12        1.55      2.86   49.20
## 16 0.000   0.044      0.03         4.20        2.78      2.74   54.54
## 17 0.000   0.068      0.02         4.56        3.06      2.97   61.50
## 18 0.000   0.057      0.03         3.84        2.64      2.86   58.02
## 19 0.041   0.303      0.09         5.39        1.27      3.40   60.43
## 20 0.071   0.000      0.10         5.47        1.31      3.54   63.67
## 21 0.107   0.000      0.19         4.84        1.11      3.47   62.05
## 22 0.480   0.000      1.36         2.88        0.64      5.40   90.64
## 23 0.453   0.000      1.46         2.99        0.59      5.54   92.33
## 24 0.545   0.000      1.27         2.78        0.50      5.47   91.48
## 25 0.017   0.039      0.14         4.56        0.94      2.12   12.85
## 26 0.003   0.036      0.12         4.86        0.99      2.39   14.11
## 27 0.044   0.011      0.02         4.23        0.93      2.25   13.48
## 28 0.000   0.133      0.02         2.81        1.89      1.74   10.60
## 29 0.000   0.132      0.03         2.59        1.92      1.66   10.20
## 30 0.000   0.136      0.21         2.26        1.75      1.70   10.40
## 31 0.119   0.237      0.08         4.42        0.96      2.99   35.55
## 32 0.099   0.296      0.09         4.75        1.02      3.11   38.00
## 33 0.104   0.231      0.12         4.03        0.99      3.05   36.77
## 34 0.000   0.258      0.71         3.98        0.04      3.11   26.50
## 35 0.038   0.237      0.72         3.90        0.04      2.97   24.55
## 36 0.106   0.160      0.30         3.05        0.43      3.04   25.52
## 37 0.000   0.020      0.08         5.43        0.80      0.00    5.04
## 38 0.005   0.013      0.08         5.61        0.86      0.00    5.43
## 39 0.000   0.019      0.17         5.76        0.95      0.00    5.24
## 40 0.000   0.155      0.03         2.15        1.35      0.00    4.95
## 41 0.000   0.155      0.03         2.35        1.18      0.00    4.32
## 42 0.000   0.149      0.17         2.46        1.43      0.00    4.63
## 43 0.013   0.273      0.03         4.80        0.80      2.74   19.43
## 44 0.114   0.245      0.03         4.93        0.83      2.78   19.55
## 45 0.088   0.002      0.03         4.25        0.26      2.76   19.49
## 46 0.013   0.391      0.43         4.67        0.05      2.57   26.10
## 47 0.022   0.021      0.46         4.87        0.05      2.64   26.31
## 48 0.123   0.000      0.12         3.85        0.23      2.61   26.20
## 49 0.002   0.043      0.17         2.74        0.30      0.00    3.85
## 50 0.004   0.049      0.18         3.13        0.32      0.00    4.30
## 51 0.000   0.021      0.07         2.65        0.08      0.00    4.08
## 52 0.000   0.113      0.27         2.03        0.76      0.00    3.12
## 53 0.000   0.126      0.31         2.02        0.78      0.00    3.35
## 54 0.000   0.096      0.27         2.33        0.85      0.00    3.23
## 55 0.104   0.000      0.08         3.94        6.46      0.00    5.57
## 56 0.148   0.000      0.08         3.91        7.14      0.00    5.85
## 57 0.111   0.000      0.22         3.93        6.84      0.00    5.71
## 58 0.078   0.099      0.08         3.72        0.04      2.49   26.70
## 59 0.171   0.000      0.09         4.11        0.04      2.59   29.69
## 60 0.144   0.000      0.31         4.99        0.29      2.54   28.20
##    Glucose Fructose TotalSugars Scopoletin PolyPhenol Ascorbic Ascorbate
## 1    34.17    24.70      114.12      28.12       4.49     0.53      1.80
## 2    37.71    27.41      114.12      23.85       4.52     0.50      1.24
## 3    35.94    26.06      114.12      25.98       4.71     0.59      0.92
## 4    14.94    13.23       80.37      15.91       4.53     0.69     13.53
## 5    16.02    14.15       80.37      21.27       4.15     0.42     14.18
## 6    15.48    13.69       80.37      18.59       4.04     0.82     11.02
## 7    77.81    65.59      248.37      64.95       3.66     0.25      2.70
## 8    86.72    74.16      248.37      63.56       3.77     0.25      3.42
## 9    82.26    69.87      248.37      64.25       3.74     0.38      2.34
## 10   53.83    52.14      226.99      83.91       4.44     0.97     13.71
## 11   57.99    56.24      226.99      99.01       3.76     0.91     14.05
## 12   55.91    54.19      226.99      91.46       4.37     0.02     14.28
## 13   29.02    26.10      108.69      46.17       6.28     0.33     26.06
## 14   30.69    27.46      108.69      44.63       6.10     3.27     25.80
## 15   29.86    26.78      108.69      45.40       6.10     2.22     26.09
## 16   29.46    25.72      117.18     116.53       3.59     0.80      2.92
## 17   31.01    26.43      117.18     133.25       3.72     0.50      4.51
## 18   30.23    26.07      117.18     124.89       3.60     0.88      3.80
## 19   44.95    41.82      153.98      78.13       3.32     1.27     34.37
## 20   46.53    43.62      153.98      85.50       3.50     1.25     34.34
## 21   45.74    42.72      153.98      81.81       3.38     1.22     34.26
## 22   86.10    80.18      265.50      95.65       3.22     2.30     31.09
## 23   88.49    82.32      265.50      88.56       3.24     2.24     31.56
## 24   87.30    81.25      265.50      92.11       3.18     2.54     30.50
## 25   65.98    66.75      150.79     122.25       5.76     1.55     27.77
## 26   68.25    69.13      150.79     119.35       5.64     1.44     28.25
## 27   67.11    67.94      150.79     120.80       5.70     1.27     27.67
## 28   24.98    31.22       68.94      47.21       4.29     0.97     19.94
## 29   25.46    32.02       68.94      49.13       4.17     0.72     20.53
## 30   25.22    31.62       68.94      48.17       4.31     0.20     20.13
## 31   79.24    81.15      206.15     121.66       4.56     1.02     19.55
## 32   84.92    87.34      206.15     126.14       4.52     0.97     19.20
## 33   82.08    84.25      206.15     123.90       4.55     1.17     19.56
## 34  118.94   126.30      272.16     104.46       3.58     2.44     44.82
## 35  116.76   125.18      272.16      85.53       3.64     2.71     44.37
## 36  117.85   125.74      272.16      95.00       3.71     2.14     44.71
## 37   53.35    59.80      123.24     126.56       3.99     3.82     27.73
## 38   57.84    65.01      123.24     125.07       3.96     3.80     29.25
## 39   55.60    62.41      123.24     125.81       3.77     3.08     27.75
## 40   17.89    24.44       45.01     102.16       5.12     1.77     16.45
## 41   16.30    22.13       45.01      61.43       5.15     1.50     15.50
## 42   17.09    23.28       45.01      81.80       5.04     2.20     16.15
## 43   90.05    98.08      213.81     214.29       2.93     1.08      7.74
## 44   93.48   101.51      213.81     213.71       3.06     1.00      8.19
## 45   91.77    99.80      213.81     214.00       2.95     1.03      7.57
## 46  100.38   114.99      248.43     175.80       3.69     2.47     27.74
## 47  104.31   119.56      248.43     212.11       3.74     2.66     25.82
## 48  102.35   117.27      248.43     193.96       3.67     2.79     25.81
## 49   17.36    17.64       40.62      68.60       6.36     2.60      6.67
## 50   18.92    19.15       40.62      64.69       6.68     2.58      6.82
## 51   18.14    18.40       40.62      66.64       6.46     2.84      7.71
## 52    9.26    13.21       26.05      54.18       3.80     2.55    119.18
## 53    9.55    13.61       26.05      55.69       3.82     2.60    119.18
## 54    9.40    13.41       26.05      54.94       3.84     2.60    118.60
## 55   41.34    43.91       92.59     100.10       5.05     1.58     71.71
## 56   43.12    45.40       92.59      96.10       5.11     1.50     74.24
## 57   42.23    44.65       92.59      98.10       4.95     0.92     75.24
## 58   83.17   104.93      229.99     218.10       6.44     3.02      8.91
## 59   93.14   117.26      229.99     228.06       6.50     3.05      8.48
## 60   88.16   111.09      229.99     223.08       6.66     3.54      9.74
##    Guaiacol Tocopherol Proteins PPDscores factor
## 1      0.15       5.80    60.64      0.00    SAN
## 2      0.16       5.26    60.74      0.00    SAN
## 3      0.10       5.67    60.64      0.00    SAN
## 4      0.15       0.29    39.29      0.00   SAN3
## 5      0.23       0.54    39.39      0.00   SAN3
## 6      0.20       0.34    39.29      0.00   SAN3
## 7      0.15       0.48    43.57      0.00   SAN5
## 8      0.13       0.28    43.67      0.00   SAN5
## 9      0.14       0.41    43.57      0.00   SAN5
## 10     0.23       0.22    37.36      0.00   SAN8
## 11     0.21       0.20    37.46      0.00   SAN8
## 12     0.21       0.22    37.36      0.00   SAN8
## 13     1.33       0.52    66.86     10.02  SAN11
## 14     1.33       0.69    66.96      0.00  SAN11
## 15     1.43       1.09    66.86     27.86  SAN11
## 16     1.39       0.34    55.79     72.98    ORI
## 17     1.27       0.37    55.89     10.02    ORI
## 18     1.37       0.24    55.79     40.25    ORI
## 19     1.08       1.89    55.36     16.75   ORI3
## 20     1.17       1.64    55.46      0.00   ORI3
## 21     0.98       2.81    55.36     33.99   ORI3
## 22     0.72       0.15    56.21     60.25   ORI5
## 23     0.80       0.22    56.31     10.02   ORI5
## 24     0.80       0.38    56.21     47.62   ORI5
## 25     0.51       4.11    82.79     72.98   ORI8
## 26     0.54       4.51    82.89     20.14   ORI8
## 27     0.52       3.50    82.79     58.24   ORI8
## 28     1.61       0.23    69.14    157.08  ORI11
## 29     1.58       0.23    69.24     41.15  ORI11
## 30     1.58       0.22    69.14     67.51  ORI11
## 31     0.24       0.26   110.50     77.54    IAC
## 32     0.24       0.20   110.60     20.14    IAC
## 33     0.23       0.31   110.50     67.51    IAC
## 34     0.84       0.23    52.57     98.51   IAC3
## 35     0.82       0.21    52.67     16.75   IAC3
## 36     0.82       0.26    52.57     67.51   IAC3
## 37     1.29       2.22    65.71     92.73   IAC5
## 38     1.25       1.52    65.81     27.00   IAC5
## 39     1.28       2.43    65.71     78.71   IAC5
## 40     5.07       3.53    21.50    157.08   IAC8
## 41     5.07       3.64    21.65     60.25   IAC8
## 42     5.07       3.68    21.50     88.67   IAC8
## 43     1.94       0.35    57.43     92.73  IAC11
## 44     1.77       0.30    57.53     30.47  IAC11
## 45     1.81       0.42    57.43     83.56  IAC11
## 46     3.16       0.21    80.00    131.20    BRA
## 47     3.13       0.25    80.11     37.55    BRA
## 48     3.05       0.29    80.00     87.37    BRA
## 49     3.34       0.47    36.14    104.85   BRA3
## 50     3.45       0.50    36.24     37.55   BRA3
## 51     3.95       0.30    36.14     90.01   BRA3
## 52     6.42       0.20    13.00    157.08   BRA5
## 53     6.58       0.27    13.22     72.98   BRA5
## 54     6.78       0.28    13.00     97.02   BRA5
## 55     3.54       0.42    18.21    111.98   BRA8
## 56     3.39       0.31    18.31     52.36   BRA8
## 57     3.41       0.32    18.21     98.51   BRA8
## 58     1.12       3.28    27.29    131.20  BRA11
## 59     1.24       1.90    27.39     56.25  BRA11
## 60     1.16       2.50    27.29    100.04  BRA11
```

```
Tabela1<-TabelaFinalTese2014[-c(3:20,31:32)]
head(Tabela1,5)
```

```
##   Sample  Days Glucose Fructose TotalSugars Scopoletin PolyPhenol Ascorbic
## 1    SAN Fresh   34.17    24.70      114.12      28.12       4.49     0.53
## 2    SAN Fresh   37.71    27.41      114.12      23.85       4.52     0.50
## 3    SAN Fresh   35.94    26.06      114.12      25.98       4.71     0.59
## 4    ORI Fresh   14.94    13.23       80.37      15.91       4.53     0.69
## 5    ORI Fresh   16.02    14.15       80.37      21.27       4.15     0.42
##   Ascorbate Guaiacol Tocopherol Proteins
## 1      1.80     0.15       5.80    60.64
## 2      1.24     0.16       5.26    60.74
## 3      0.92     0.10       5.67    60.64
## 4     13.53     0.15       0.29    39.29
## 5     14.18     0.23       0.54    39.39
```

```
###two-way anova of the variables 
gluco<-Tabela1[1:3]  ##glucose
frut<-Tabela1[-c(3,5:12)]  ##frutose
tsug<-Tabela1[-c(3:4,6:12)]  ##total sugars
scopo<-Tabela1[-c(3:5,7:12)] ##scopoletin
poli<-Tabela1[-c(3:6,8:12)]  ##poliphenol oxidase
abic<-Tabela1[-c(3:7, 9:12)]  ##ascorbic acid
abate<-Tabela1[-c(3:8,10:12)] ##ascorbate peroxidase
gua<-Tabela1[-c(3:9, 11:12)]  ##guaiacol
toco<-Tabela1[-c(3:10,12)]  ##tocopherol
pro<-Tabela1[-c(3:11)]  ##proteins

require(easyanova)
gluco1<-ea2(gluco, design=1)
```

```
frut1<-ea2(frut, design=1)
```

```
tsug1<-ea2(tsug, design=1)
```

```
scopo1<-ea2(scopo, design=1)
```

```
poli1<-ea2(poli, design=1)
```

```
abic1<-ea2(abic, design=1)
```

```
abate1<-ea2(abate, design=1)
```

```
gua1<-ea2(gua, design=1)
```

```
toco1<-ea2(toco, design=1)
```

```
pro1<-ea2(pro, design=1)
```

```
gluco1$"Adjusted means (factor 2 in levels of factor 1)"
```

```
## $`factor_2 in  BRA`
##    treatment adjusted.mean standard.error tukey snk duncan t scott_knott
## 9   BRA.Day5      117.8500         1.1568     a   a      a a           a
## 13  BRA.Day8      102.3467         1.1568     b   b      b b           b
## 1  BRA.Day11       88.1567         1.1568     c   c      c c           c
## 5   BRA.Day3       87.2967         1.1568     c   c      c c           c
## 17 BRA.Fresh       55.9100         1.1568     d   d      d d           d
## 
## $`factor_2 in  IAC`
##    treatment adjusted.mean standard.error tukey snk duncan t scott_knott
## 14  IAC.Day8       91.7667         1.1568     a   a      a a           a
## 18 IAC.Fresh       82.2633         1.1568     b   b      b b           b
## 10  IAC.Day5       82.0800         1.1568     b   b      b b           b
## 6   IAC.Day3       45.7400         1.1568     c   c      c c           c
## 2  IAC.Day11       42.2300         1.1568     c   d      d d           d
## 
## $`factor_2 in  ORI`
##    treatment adjusted.mean standard.error tukey snk duncan t scott_knott
## 7   ORI.Day3       30.2333         1.1568     a   a      a a           a
## 11  ORI.Day5       25.2200         1.1568     b   b      b b           b
## 15  ORI.Day8       17.0933         1.1568     c   c      c c           c
## 19 ORI.Fresh       15.4800         1.1568     c   c      c c           c
## 3  ORI.Day11        9.4033         1.1568     d   d      d d           d
## 
## $`factor_2 in  SAN`
##    treatment adjusted.mean standard.error tukey snk duncan t scott_knott
## 12  SAN.Day5       67.1133         1.1568     a   a      a a           a
## 16  SAN.Day8       55.5967         1.1568     b   b      b b           b
## 20 SAN.Fresh       35.9400         1.1568     c   c      c c           c
## 8   SAN.Day3       29.8567         1.1568     d   d      d d           d
## 4  SAN.Day11       18.1400         1.1568     e   e      e e           e
```

```
frut1$"Adjusted means (factor 2 in levels of factor 1)"
```

```
## $`factor_2 in  BRA`
##    treatment adjusted.mean standard.error tukey snk duncan t scott_knott
## 9   BRA.Day5      125.7400         1.2522     a   a      a a           a
## 13  BRA.Day8      117.2733         1.2522     b   b      b b           b
## 1  BRA.Day11      111.0933         1.2522     c   c      c c           c
## 5   BRA.Day3       81.2500         1.2522     d   d      d d           d
## 17 BRA.Fresh       54.1900         1.2522     e   e      e e           e
## 
## $`factor_2 in  IAC`
##    treatment adjusted.mean standard.error tukey snk duncan t scott_knott
## 14  IAC.Day8       99.7967         1.2522     a   a      a a           a
## 10  IAC.Day5       84.2467         1.2522     b   b      b b           b
## 18 IAC.Fresh       69.8733         1.2522     c   c      c c           c
## 2  IAC.Day11       44.6533         1.2522     d   d      d d           d
## 6   IAC.Day3       42.7200         1.2522     d   d      d d           d
## 
## $`factor_2 in  ORI`
##    treatment adjusted.mean standard.error tukey snk duncan t scott_knott
## 11  ORI.Day5       31.6200         1.2522     a   a      a a           a
## 7   ORI.Day3       26.0733         1.2522     b   b      b b           b
## 15  ORI.Day8       23.2833         1.2522     b   b      b b           b
## 19 ORI.Fresh       13.6900         1.2522     c   c      c c           c
## 3  ORI.Day11       13.4100         1.2522     c   c      c c           c
## 
## $`factor_2 in  SAN`
##    treatment adjusted.mean standard.error tukey snk duncan t scott_knott
## 12  SAN.Day5       67.9400         1.2522     a   a      a a           a
## 16  SAN.Day8       62.4067         1.2522     b   b      b b           b
## 8   SAN.Day3       26.7800         1.2522     c   c      c c           c
## 20 SAN.Fresh       26.0567         1.2522     c   c      c c           c
## 4  SAN.Day11       18.3967         1.2522     d   d      d d           d
```

```
tsug1$"Adjusted means (factor 2 in levels of factor 1)"
```

```
## $`factor_2 in  BRA`
##    treatment adjusted.mean standard.error tukey snk duncan t scott_knott
## 9   BRA.Day5        272.16              0     a   a      a a           a
## 5   BRA.Day3        265.50              0     b   b      b b           b
## 13  BRA.Day8        248.43              0     c   c      c c           c
## 1  BRA.Day11        229.99              0     d   d      d d           d
## 17 BRA.Fresh        226.99              0     e   e      e e           e
## 
## $`factor_2 in  IAC`
##    treatment adjusted.mean standard.error tukey snk duncan t scott_knott
## 18 IAC.Fresh        248.37              0     a   a      a a           a
## 14  IAC.Day8        213.81              0     b   b      b b           b
## 10  IAC.Day5        206.15              0     c   c      c c           c
## 6   IAC.Day3        153.98              0     d   d      d d           d
## 2  IAC.Day11         92.59              0     e   e      e e           e
## 
## $`factor_2 in  ORI`
##    treatment adjusted.mean standard.error tukey snk duncan t scott_knott
## 7   ORI.Day3        117.18              0     a   a      a a           a
## 19 ORI.Fresh         80.37              0     b   b      b b           b
## 11  ORI.Day5         68.94              0     c   c      c c           c
## 15  ORI.Day8         45.01              0     d   d      d d           d
## 3  ORI.Day11         26.05              0     e   e      e e           e
## 
## $`factor_2 in  SAN`
##    treatment adjusted.mean standard.error tukey snk duncan t scott_knott
## 12  SAN.Day5        150.79              0     a   a      a a           a
## 16  SAN.Day8        123.24              0     b   b      b b           b
## 20 SAN.Fresh        114.12              0     c   c      c c           c
## 8   SAN.Day3        108.69              0     d   d      d d           d
## 4  SAN.Day11         40.62              0     e   e      e e           e
```

```
scopo1$"Adjusted means (factor 2 in levels of factor 1)"
```

```
## $`factor_2 in  BRA`
##    treatment adjusted.mean standard.error tukey snk duncan t scott_knott
## 1  BRA.Day11      223.0800         4.1666     a   a      a a           a
## 13  BRA.Day8      193.9567         4.1666     b   b      b b           b
## 9   BRA.Day5       94.9967         4.1666     c   c      c c           c
## 5   BRA.Day3       92.1067         4.1666     c   c      c c           c
## 17 BRA.Fresh       91.4600         4.1666     c   c      c c           c
## 
## $`factor_2 in  IAC`
##    treatment adjusted.mean standard.error tukey snk duncan t scott_knott
## 14  IAC.Day8      214.0000         4.1666     a   a      a a           a
## 10  IAC.Day5      123.9000         4.1666     b   b      b b           b
## 2  IAC.Day11       98.1000         4.1666     c   c      c c           c
## 6   IAC.Day3       81.8133         4.1666     c   d      d d           d
## 18 IAC.Fresh       64.2533         4.1666     d   e      e e           e
## 
## $`factor_2 in  ORI`
##    treatment adjusted.mean standard.error tukey snk duncan t scott_knott
## 7   ORI.Day3      124.8900         4.1666     a   a      a a           a
## 15  ORI.Day8       81.7967         4.1666     b   b      b b           b
## 3  ORI.Day11       54.9367         4.1666     c   c      c c           c
## 11  ORI.Day5       48.1700         4.1666     c   c      c c           c
## 19 ORI.Fresh       18.5900         4.1666     d   d      d d           d
## 
## $`factor_2 in  SAN`
##    treatment adjusted.mean standard.error tukey snk duncan t scott_knott
## 16  SAN.Day8      125.8133         4.1666     a   a      a a           a
## 12  SAN.Day5      120.8000         4.1666     a   a      a a           a
## 4  SAN.Day11       66.6433         4.1666     b   b      b b           b
## 8   SAN.Day3       45.4000         4.1666     c   c      c c           c
## 20 SAN.Fresh       25.9833         4.1666     d   d      d d           d
```

```
poli1$"Adjusted means (factor 2 in levels of factor 1)"
```

```
## $`factor_2 in  BRA`
##    treatment adjusted.mean standard.error tukey snk duncan t scott_knott
## 1  BRA.Day11        6.5333         0.0745     a   a      a a           a
## 17 BRA.Fresh        4.1900         0.0745     b   b      b b           b
## 13  BRA.Day8        3.7000         0.0745     c   c      c c           c
## 9   BRA.Day5        3.6433         0.0745     c   c      c c           c
## 5   BRA.Day3        3.2133         0.0745     d   d      d d           d
## 
## $`factor_2 in  IAC`
##    treatment adjusted.mean standard.error tukey snk duncan t scott_knott
## 2  IAC.Day11        5.0367         0.0745     a   a      a a           a
## 10  IAC.Day5        4.5433         0.0745     b   b      b b           b
## 18 IAC.Fresh        3.7233         0.0745     c   c      c c           c
## 6   IAC.Day3        3.4000         0.0745     d   d      d d           d
## 14  IAC.Day8        2.9800         0.0745     e   e      e e           e
## 
## $`factor_2 in  ORI`
##    treatment adjusted.mean standard.error tukey snk duncan t scott_knott
## 15  ORI.Day8        5.1033         0.0745     a   a      a a           a
## 11  ORI.Day5        4.2567         0.0745     b   b      b b           b
## 19 ORI.Fresh        4.2400         0.0745     b   b      b b           b
## 3  ORI.Day11        3.8200         0.0745     c   c      c c           c
## 7   ORI.Day3        3.6367         0.0745     c   c      c c           c
## 
## $`factor_2 in  SAN`
##    treatment adjusted.mean standard.error tukey snk duncan t scott_knott
## 4  SAN.Day11        6.5000         0.0745     a   a      a a           a
## 8   SAN.Day3        6.1600         0.0745     b   b      b b           b
## 12  SAN.Day5        5.7000         0.0745     c   c      c c           c
## 20 SAN.Fresh        4.5733         0.0745     d   d      d d           d
## 16  SAN.Day8        3.9067         0.0745     e   e      e e           e
```

```
abic1$"Adjusted means (factor 2 in levels of factor 1)"
```

```
## $`factor_2 in  BRA`
##    treatment adjusted.mean standard.error tukey snk duncan  t scott_knott
## 1  BRA.Day11        3.2033         0.2398     a   a      a  a           a
## 13  BRA.Day8        2.6400         0.2398     a   a     ab ab           b
## 9   BRA.Day5        2.4300         0.2398     a   a      b  b           b
## 5   BRA.Day3        2.3600         0.2398     a   a      b  b           b
## 17 BRA.Fresh        0.6333         0.2398     b   b      c  c           c
## 
## $`factor_2 in  IAC`
##    treatment adjusted.mean standard.error tukey snk duncan t scott_knott
## 2  IAC.Day11        1.3333         0.2398     a   a      a a           a
## 6   IAC.Day3        1.2467         0.2398    ab   a      a a           a
## 10  IAC.Day5        1.0533         0.2398    ab  ab      a a           a
## 14  IAC.Day8        1.0367         0.2398    ab   a      a a           a
## 18 IAC.Fresh        0.2933         0.2398     b   b      b b           b
## 
## $`factor_2 in  ORI`
##    treatment adjusted.mean standard.error tukey snk duncan t scott_knott
## 3  ORI.Day11        2.5833         0.2398     a   a      a a           a
## 15  ORI.Day8        1.8233         0.2398     a   b      b b           b
## 7   ORI.Day3        0.7267         0.2398     b   c      c c           c
## 19 ORI.Fresh        0.6433         0.2398     b   c      c c           c
## 11  ORI.Day5        0.6300         0.2398     b   c      c c           c
## 
## $`factor_2 in  SAN`
##    treatment adjusted.mean standard.error tukey snk duncan t scott_knott
## 16  SAN.Day8        3.5667         0.2398     a   a      a a           a
## 4  SAN.Day11        2.6733         0.2398    ab   b      b b           b
## 8   SAN.Day3        1.9400         0.2398    bc   c      c c           c
## 12  SAN.Day5        1.4200         0.2398    cd   c      c c           c
## 20 SAN.Fresh        0.5400         0.2398     d   d      d d           d
```

```
abate1$"Adjusted means (factor 2 in levels of factor 1)"
```

```
## $`factor_2 in  BRA`
##    treatment adjusted.mean standard.error tukey snk duncan t scott_knott
## 9   BRA.Day5       44.6333         0.4297     a   a      a a           a
## 5   BRA.Day3       31.0500         0.4297     b   b      b b           b
## 13  BRA.Day8       26.4567         0.4297     c   c      c c           c
## 17 BRA.Fresh       14.0133         0.4297     d   d      d d           d
## 1  BRA.Day11        9.0433         0.4297     e   e      e e           e
## 
## $`factor_2 in  IAC`
##    treatment adjusted.mean standard.error tukey snk duncan t scott_knott
## 2  IAC.Day11       73.7300         0.4297     a   a      a a           a
## 6   IAC.Day3       34.3233         0.4297     b   b      b b           b
## 10  IAC.Day5       19.4367         0.4297     c   c      c c           c
## 14  IAC.Day8        7.8333         0.4297     d   d      d d           d
## 18 IAC.Fresh        2.8200         0.4297     e   e      e e           e
## 
## $`factor_2 in  ORI`
##    treatment adjusted.mean standard.error tukey snk duncan t scott_knott
## 3  ORI.Day11      118.9867         0.4297     a   a      a a           a
## 11  ORI.Day5       20.2000         0.4297     b   b      b b           b
## 15  ORI.Day8       16.0333         0.4297     c   c      c c           c
## 19 ORI.Fresh       12.9100         0.4297     d   d      d d           d
## 7   ORI.Day3        3.7433         0.4297     e   e      e e           e
## 
## $`factor_2 in  SAN`
##    treatment adjusted.mean standard.error tukey snk duncan t scott_knott
## 16  SAN.Day8       28.2433         0.4297     a   a      a a           a
## 12  SAN.Day5       27.8967         0.4297     a   a      a a           a
## 8   SAN.Day3       25.9833         0.4297     b   b      b b           b
## 4  SAN.Day11        7.0667         0.4297     c   c      c c           c
## 20 SAN.Fresh        1.3200         0.4297     d   d      d d           d
```

```
gua1$"Adjusted means (factor 2 in levels of factor 1)"
```

```
## $`factor_2 in  BRA`
##    treatment adjusted.mean standard.error tukey snk duncan t scott_knott
## 13  BRA.Day8        3.1133         0.0551     a   a      a a           a
## 1  BRA.Day11        1.1733         0.0551     b   b      b b           b
## 9   BRA.Day5        0.8267         0.0551     c   c      c c           c
## 5   BRA.Day3        0.7733         0.0551     c   c      c c           c
## 17 BRA.Fresh        0.2167         0.0551     d   d      d d           d
## 
## $`factor_2 in  IAC`
##    treatment adjusted.mean standard.error tukey snk duncan t scott_knott
## 2  IAC.Day11        3.4467         0.0551     a   a      a a           a
## 14  IAC.Day8        1.8400         0.0551     b   b      b b           b
## 6   IAC.Day3        1.0767         0.0551     c   c      c c           c
## 10  IAC.Day5        0.2367         0.0551     d   d      d d           d
## 18 IAC.Fresh        0.1400         0.0551     d   d      d d           d
## 
## $`factor_2 in  ORI`
##    treatment adjusted.mean standard.error tukey snk duncan t scott_knott
## 3  ORI.Day11        6.5933         0.0551     a   a      a a           a
## 15  ORI.Day8        5.0700         0.0551     b   b      b b           b
## 11  ORI.Day5        1.5900         0.0551     c   c      c c           c
## 7   ORI.Day3        1.3433         0.0551     d   d      d d           d
## 19 ORI.Fresh        0.1933         0.0551     e   e      e e           e
## 
## $`factor_2 in  SAN`
##    treatment adjusted.mean standard.error tukey snk duncan t scott_knott
## 4  SAN.Day11        3.5800         0.0551     a   a      a a           a
## 8   SAN.Day3        1.3633         0.0551     b   b      b b           b
## 16  SAN.Day8        1.2733         0.0551     b   b      b b           b
## 12  SAN.Day5        0.5233         0.0551     c   c      c c           c
## 20 SAN.Fresh        0.1367         0.0551     d   d      d d           d
```

```
toco1$"Adjusted means (factor 2 in levels of factor 1)"
```

```
## $`factor_2 in  BRA`
##    treatment adjusted.mean standard.error tukey snk duncan t scott_knott
## 1  BRA.Day11        2.5600         0.1627     a   a      a a           a
## 5   BRA.Day3        0.2500         0.1627     b   b      b b           b
## 13  BRA.Day8        0.2500         0.1627     b   b      b b           b
## 9   BRA.Day5        0.2333         0.1627     b   b      b b           b
## 17 BRA.Fresh        0.2133         0.1627     b   b      b b           b
## 
## $`factor_2 in  IAC`
##    treatment adjusted.mean standard.error tukey snk duncan t scott_knott
## 6   IAC.Day3        2.1133         0.1627     a   a      a a           a
## 18 IAC.Fresh        0.3900         0.1627     b   b      b b           b
## 14  IAC.Day8        0.3567         0.1627     b   b      b b           b
## 2  IAC.Day11        0.3500         0.1627     b   b      b b           b
## 10  IAC.Day5        0.2567         0.1627     b   b      b b           b
## 
## $`factor_2 in  ORI`
##    treatment adjusted.mean standard.error tukey snk duncan t scott_knott
## 15  ORI.Day8        3.6167         0.1627     a   a      a a           a
## 19 ORI.Fresh        0.3900         0.1627     b   b      b b           b
## 7   ORI.Day3        0.3167         0.1627     b   b      b b           b
## 3  ORI.Day11        0.2500         0.1627     b   b      b b           b
## 11  ORI.Day5        0.2267         0.1627     b   b      b b           b
## 
## $`factor_2 in  SAN`
##    treatment adjusted.mean standard.error tukey snk duncan t scott_knott
## 20 SAN.Fresh        5.5767         0.1627     a   a      a a           a
## 12  SAN.Day5        4.0400         0.1627     b   b      b b           b
## 16  SAN.Day8        2.0567         0.1627     c   c      c c           c
## 8   SAN.Day3        0.7667         0.1627     d   d      d d           d
## 4  SAN.Day11        0.4233         0.1627     d   d      d d           d
```

```
pro1$"Adjusted means (factor 2 in levels of factor 1)"
```

```
## $`factor_2 in  BRA`
##    treatment adjusted.mean standard.error tukey snk duncan t scott_knott
## 13  BRA.Day8       80.0367         0.0375     a   a      a a           a
## 5   BRA.Day3       56.2433         0.0375     b   b      b b           b
## 9   BRA.Day5       52.6033         0.0375     c   c      c c           c
## 17 BRA.Fresh       37.3933         0.0375     d   d      d d           d
## 1  BRA.Day11       27.3233         0.0375     e   e      e e           e
## 
## $`factor_2 in  IAC`
##    treatment adjusted.mean standard.error tukey snk duncan t scott_knott
## 10  IAC.Day5      110.5333         0.0375     a   a      a a           a
## 14  IAC.Day8       57.4633         0.0375     b   b      b b           b
## 6   IAC.Day3       55.3933         0.0375     c   c      c c           c
## 18 IAC.Fresh       43.6033         0.0375     d   d      d d           d
## 2  IAC.Day11       18.2433         0.0375     e   e      e e           e
## 
## $`factor_2 in  ORI`
##    treatment adjusted.mean standard.error tukey snk duncan t scott_knott
## 11  ORI.Day5       69.1733         0.0375     a   a      a a           a
## 7   ORI.Day3       55.8233         0.0375     b   b      b b           b
## 19 ORI.Fresh       39.3233         0.0375     c   c      c c           c
## 15  ORI.Day8       21.5500         0.0375     d   d      d d           d
## 3  ORI.Day11       13.0733         0.0375     e   e      e e           e
## 
## $`factor_2 in  SAN`
##    treatment adjusted.mean standard.error tukey snk duncan t scott_knott
## 12  SAN.Day5       82.8233         0.0375     a   a      a a           a
## 8   SAN.Day3       66.8933         0.0375     b   b      b b           b
## 16  SAN.Day8       65.7433         0.0375     c   c      c c           c
## 20 SAN.Fresh       60.6733         0.0375     d   d      d d           d
## 4  SAN.Day11       36.1733         0.0375     e   e      e e           e
```

### OLS REGRESSION MODELS

```
setwd("C:/Users/Virgílio/Desktop/PASTAS DO DESKTOP/ascorbato peroxidase")
require(lme4)
require(arm) 
data1<-TabelaFinalTese2014[3:31]
data2<-TabelaFinalTese2014[2:31]   ##include days
data3<-TabelaFinalTese2014[1:31]   ##include cultivars

Model1 <- glm(PPDscores ~ ., data = data1)  ##usando todas variaveis (menos factors)
display(Model1)
```

```
## glm(formula = PPDscores ~ ., data = data1)
##                  coef.est  coef.se  
## (Intercept)        -191.09    221.92
## Phenolics            -0.13      0.12
## Flavonoids            0.01      0.03
## Carotenoids          -1.86      5.05
## Anthocyanins          0.88      1.55
## Totalcyanide      20536.50  11608.23
## AcetoneCyano     -20516.50  11607.22
## Linamarin        -20535.11  11608.23
## Linamarase           12.63     24.72
## HydrogenPeroxide     -0.13      0.39
## Catalase              0.40      0.24
## TotalSOD            100.81    154.48
## MnSOD               -28.89    118.24
## CuZnSOD             -50.24    177.59
## MalicAcid            20.47     53.67
## SuccinicAcid        -20.66     13.98
## FumaricAcid           4.80     12.96
## Raffinose            40.17     35.45
## Sucrose              -4.08      1.79
## Glucose              -2.64      5.27
## Fructose             -2.17      5.12
## TotalSugars           1.83      0.98
## Scopoletin            0.35      0.39
## PolyPhenol          -18.88     20.79
## Ascorbic              8.41     11.72
## Ascorbate            -0.70      0.87
## Guaiacol             11.72     14.70
## Tocopherol            5.05      6.44
## Proteins              1.01      1.31
## ---
##   n = 60, k = 29
##   residual deviance = 31747.9, null deviance = 122979.3 (difference = 91231.5)
##   overdispersion parameter = 1024.1
##   residual sd is sqrt(overdispersion) = 32.00
```

```
AIC(Model1)     ## 606.5 ##akaike information criterion (quality of statistical model= must minor in testing our model, relative to information lost)
```

```
## [1] 606.5468
```

```
Model2 <- glm(PPDscores ~ ., data = data2)
display(Model2)
```

```
## glm(formula = PPDscores ~ ., data = data2)
##                  coef.est  coef.se  
## (Intercept)        1221.55    487.82
## DaysDay3           -335.27    249.60
## DaysDay5           -207.14    153.34
## DaysDay8           -168.17     66.25
## DaysFresh          -559.66    292.38
## Phenolics            -0.30      0.15
## Flavonoids            0.01      0.03
## Carotenoids          -7.82      5.16
## Anthocyanins          0.11      1.49
## Totalcyanide      25857.43  11279.45
## AcetoneCyano     -25870.54  11282.13
## Linamarin        -25858.80  11279.72
## Linamarase          -57.77     33.91
## HydrogenPeroxide     -1.29      1.02
## Catalase             -0.22      0.32
## TotalSOD             64.63    141.90
## MnSOD               -23.94    141.42
## CuZnSOD             -25.75    166.41
## MalicAcid           -66.72     55.25
## SuccinicAcid        -16.95     14.30
## FumaricAcid           6.36     18.04
## Raffinose            -2.34     49.62
## Sucrose              -0.19      2.13
## Glucose               3.05      6.07
## Fructose             -8.89      5.93
## TotalSugars           3.00      0.97
## Scopoletin            0.79      0.44
## PolyPhenol          -21.59     21.63
## Ascorbic             12.06     11.40
## Ascorbate             0.70      0.90
## Guaiacol            -18.51     17.02
## Tocopherol           -1.06      6.33
## Proteins             -2.36      1.63
## ---
##   n = 60, k = 33
##   residual deviance = 22275.3, null deviance = 122979.3 (difference = 100704.0)
##   overdispersion parameter = 825.0
##   residual sd is sqrt(overdispersion) = 28.72
```

```
AIC(Model2)    ## 593.3  something was improved
```

```
## [1] 593.2861
```

```
Model3 <- glm(PPDscores ~ ., data = data3)
display(Model3)
```

```
## glm(formula = PPDscores ~ ., data = data3)
##                  coef.est  coef.se  
## (Intercept)        1234.64    511.13
## SampleIAC             1.24    159.89
## SampleORI           -90.11    240.62
## SampleSAN           -93.52    271.83
## DaysDay3           -293.09    328.20
## DaysDay5           -128.66    211.58
## DaysDay8           -134.81    145.57
## DaysFresh          -424.97    368.41
## Phenolics            -0.28      0.17
## Flavonoids            0.03      0.04
## Carotenoids          -7.71      5.61
## Anthocyanins          0.89      1.85
## Totalcyanide      27608.13  12095.61
## AcetoneCyano     -27622.91  12099.96
## Linamarin        -27608.98  12095.58
## Linamarase          -53.52     56.65
## HydrogenPeroxide     -1.85      1.43
## Catalase             -0.14      0.36
## TotalSOD             26.60    165.57
## MnSOD                14.84    174.10
## CuZnSOD              32.73    198.99
## MalicAcid           -50.75     62.84
## SuccinicAcid        -20.31     16.98
## FumaricAcid           7.36     19.46
## Raffinose            -7.73     68.66
## Sucrose              -0.74      4.24
## Glucose               3.03      9.85
## Fructose             -9.31      9.62
## TotalSugars           2.58      1.97
## Scopoletin            1.33      0.86
## PolyPhenol          -17.72     28.31
## Ascorbic             15.01     12.51
## Ascorbate             0.86      1.22
## Guaiacol            -14.74     31.11
## Tocopherol           -4.34     10.25
## Proteins             -2.22      2.12
## ---
##   n = 60, k = 36
##   residual deviance = 21703.6, null deviance = 122979.3 (difference = 101275.7)
##   overdispersion parameter = 904.3
##   residual sd is sqrt(overdispersion) = 30.07
```

```
AIC(Model3)   ## Nothing was improved
```

```
## [1] 597.726
```

```
anova(Model1,Model2, test="F")  ## there is difference at 5%
```

```
## Analysis of Deviance Table
## 
## Model 1: PPDscores ~ Phenolics + Flavonoids + Carotenoids + Anthocyanins + 
##     Totalcyanide + AcetoneCyano + Linamarin + Linamarase + HydrogenPeroxide + 
##     Catalase + TotalSOD + MnSOD + CuZnSOD + MalicAcid + SuccinicAcid + 
##     FumaricAcid + Raffinose + Sucrose + Glucose + Fructose + 
##     TotalSugars + Scopoletin + PolyPhenol + Ascorbic + Ascorbate + 
##     Guaiacol + Tocopherol + Proteins
## Model 2: PPDscores ~ Days + Phenolics + Flavonoids + Carotenoids + Anthocyanins + 
##     Totalcyanide + AcetoneCyano + Linamarin + Linamarase + HydrogenPeroxide + 
##     Catalase + TotalSOD + MnSOD + CuZnSOD + MalicAcid + SuccinicAcid + 
##     FumaricAcid + Raffinose + Sucrose + Glucose + Fructose + 
##     TotalSugars + Scopoletin + PolyPhenol + Ascorbic + Ascorbate + 
##     Guaiacol + Tocopherol + Proteins
##   Resid. Df Resid. Dev Df Deviance      F  Pr(>F)  
## 1        31      31748                             
## 2        27      22275  4   9472.5 2.8704 0.04206 *
## ---
## Signif. codes:  0 '***' 0.001 '**' 0.01 '*' 0.05 '.' 0.1 ' ' 1
```

```
anova(Model1, Model3, test="F")## there is no difference
```

```
## Analysis of Deviance Table
## 
## Model 1: PPDscores ~ Phenolics + Flavonoids + Carotenoids + Anthocyanins + 
##     Totalcyanide + AcetoneCyano + Linamarin + Linamarase + HydrogenPeroxide + 
##     Catalase + TotalSOD + MnSOD + CuZnSOD + MalicAcid + SuccinicAcid + 
##     FumaricAcid + Raffinose + Sucrose + Glucose + Fructose + 
##     TotalSugars + Scopoletin + PolyPhenol + Ascorbic + Ascorbate + 
##     Guaiacol + Tocopherol + Proteins
## Model 2: PPDscores ~ Sample + Days + Phenolics + Flavonoids + Carotenoids + 
##     Anthocyanins + Totalcyanide + AcetoneCyano + Linamarin + 
##     Linamarase + HydrogenPeroxide + Catalase + TotalSOD + MnSOD + 
##     CuZnSOD + MalicAcid + SuccinicAcid + FumaricAcid + Raffinose + 
##     Sucrose + Glucose + Fructose + TotalSugars + Scopoletin + 
##     PolyPhenol + Ascorbic + Ascorbate + Guaiacol + Tocopherol + 
##     Proteins
##   Resid. Df Resid. Dev Df Deviance      F Pr(>F)
## 1        31      31748                          
## 2        24      21704  7    10044 1.5867 0.1873
```

```
anova(Model2,Model3,test="F")  ##THERE IS NO DIFF.
```

```
## Analysis of Deviance Table
## 
## Model 1: PPDscores ~ Days + Phenolics + Flavonoids + Carotenoids + Anthocyanins + 
##     Totalcyanide + AcetoneCyano + Linamarin + Linamarase + HydrogenPeroxide + 
##     Catalase + TotalSOD + MnSOD + CuZnSOD + MalicAcid + SuccinicAcid + 
##     FumaricAcid + Raffinose + Sucrose + Glucose + Fructose + 
##     TotalSugars + Scopoletin + PolyPhenol + Ascorbic + Ascorbate + 
##     Guaiacol + Tocopherol + Proteins
## Model 2: PPDscores ~ Sample + Days + Phenolics + Flavonoids + Carotenoids + 
##     Anthocyanins + Totalcyanide + AcetoneCyano + Linamarin + 
##     Linamarase + HydrogenPeroxide + Catalase + TotalSOD + MnSOD + 
##     CuZnSOD + MalicAcid + SuccinicAcid + FumaricAcid + Raffinose + 
##     Sucrose + Glucose + Fructose + TotalSugars + Scopoletin + 
##     PolyPhenol + Ascorbic + Ascorbate + Guaiacol + Tocopherol + 
##     Proteins
##   Resid. Df Resid. Dev Df Deviance      F Pr(>F)
## 1        27      22275                          
## 2        24      21704  3   571.71 0.2107  0.888
```

```
anova(Model1, Model2, Model3, test="F")  ##second model is the best (MINOR AIC)
```

```
## Analysis of Deviance Table
## 
## Model 1: PPDscores ~ Phenolics + Flavonoids + Carotenoids + Anthocyanins + 
##     Totalcyanide + AcetoneCyano + Linamarin + Linamarase + HydrogenPeroxide + 
##     Catalase + TotalSOD + MnSOD + CuZnSOD + MalicAcid + SuccinicAcid + 
##     FumaricAcid + Raffinose + Sucrose + Glucose + Fructose + 
##     TotalSugars + Scopoletin + PolyPhenol + Ascorbic + Ascorbate + 
##     Guaiacol + Tocopherol + Proteins
## Model 2: PPDscores ~ Days + Phenolics + Flavonoids + Carotenoids + Anthocyanins + 
##     Totalcyanide + AcetoneCyano + Linamarin + Linamarase + HydrogenPeroxide + 
##     Catalase + TotalSOD + MnSOD + CuZnSOD + MalicAcid + SuccinicAcid + 
##     FumaricAcid + Raffinose + Sucrose + Glucose + Fructose + 
##     TotalSugars + Scopoletin + PolyPhenol + Ascorbic + Ascorbate + 
##     Guaiacol + Tocopherol + Proteins
## Model 3: PPDscores ~ Sample + Days + Phenolics + Flavonoids + Carotenoids + 
##     Anthocyanins + Totalcyanide + AcetoneCyano + Linamarin + 
##     Linamarase + HydrogenPeroxide + Catalase + TotalSOD + MnSOD + 
##     CuZnSOD + MalicAcid + SuccinicAcid + FumaricAcid + Raffinose + 
##     Sucrose + Glucose + Fructose + TotalSugars + Scopoletin + 
##     PolyPhenol + Ascorbic + Ascorbate + Guaiacol + Tocopherol + 
##     Proteins
##   Resid. Df Resid. Dev Df Deviance      F Pr(>F)  
## 1        31      31748                            
## 2        27      22275  4   9472.5 2.6187 0.0602 .
## 3        24      21704  3    571.7 0.2107 0.8880  
## ---
## Signif. codes:  0 '***' 0.001 '**' 0.01 '*' 0.05 '.' 0.1 ' ' 1
```

```
setwd("C:/Users/Virgílio/Desktop/PASTAS DO DESKTOP/ascorbato peroxidase")
secMetabo<-TabelaFinalTese2014[-c(7:23,25:30,32)]
Cyanogenic<-TabelaFinalTese2014[-c(3:6,11:30,32)]
Enzymes<-TabelaFinalTese2014[-c(3:24,32)]
SugarAcids<-TabelaFinalTese2014[-c(3:15,24:30,32)]
ROS<-TabelaFinalTese2014[-c(3:10,16:30,32)]

require(plyr)
setwd("C:/Users/Virgílio/Desktop/PASTAS DO DESKTOP/ascorbato peroxidase")
modellist <- dlply(TabelaFinalTese2014[1:31], .(Sample, Days), function(x) glm(PPDscores ~ ., data = TabelaFinalTese2014[1:31]))
display(modellist[[1]])
```

```
## glm(formula = PPDscores ~ ., data = TabelaFinalTese2014[1:31])
##                  coef.est  coef.se  
## (Intercept)        1234.64    511.13
## SampleIAC             1.24    159.89
## SampleORI           -90.11    240.62
## SampleSAN           -93.52    271.83
## DaysDay3           -293.09    328.20
## DaysDay5           -128.66    211.58
## DaysDay8           -134.81    145.57
## DaysFresh          -424.97    368.41
## Phenolics            -0.28      0.17
## Flavonoids            0.03      0.04
## Carotenoids          -7.71      5.61
## Anthocyanins          0.89      1.85
## Totalcyanide      27608.13  12095.61
## AcetoneCyano     -27622.91  12099.96
## Linamarin        -27608.98  12095.58
## Linamarase          -53.52     56.65
## HydrogenPeroxide     -1.85      1.43
## Catalase             -0.14      0.36
## TotalSOD             26.60    165.57
## MnSOD                14.84    174.10
## CuZnSOD              32.73    198.99
## MalicAcid           -50.75     62.84
## SuccinicAcid        -20.31     16.98
## FumaricAcid           7.36     19.46
## Raffinose            -7.73     68.66
## Sucrose              -0.74      4.24
## Glucose               3.03      9.85
## Fructose             -9.31      9.62
## TotalSugars           2.58      1.97
## Scopoletin            1.33      0.86
## PolyPhenol          -17.72     28.31
## Ascorbic             15.01     12.51
## Ascorbate             0.86      1.22
## Guaiacol            -14.74     31.11
## Tocopherol           -4.34     10.25
## Proteins             -2.22      2.12
## ---
##   n = 60, k = 36
##   residual deviance = 21703.6, null deviance = 122979.3 (difference = 101275.7)
##   overdispersion parameter = 904.3
##   residual sd is sqrt(overdispersion) = 30.07
```

```
display(modellist[[2]])
```

```
## glm(formula = PPDscores ~ ., data = TabelaFinalTese2014[1:31])
##                  coef.est  coef.se  
## (Intercept)        1234.64    511.13
## SampleIAC             1.24    159.89
## SampleORI           -90.11    240.62
## SampleSAN           -93.52    271.83
## DaysDay3           -293.09    328.20
## DaysDay5           -128.66    211.58
## DaysDay8           -134.81    145.57
## DaysFresh          -424.97    368.41
## Phenolics            -0.28      0.17
## Flavonoids            0.03      0.04
## Carotenoids          -7.71      5.61
## Anthocyanins          0.89      1.85
## Totalcyanide      27608.13  12095.61
## AcetoneCyano     -27622.91  12099.96
## Linamarin        -27608.98  12095.58
## Linamarase          -53.52     56.65
## HydrogenPeroxide     -1.85      1.43
## Catalase             -0.14      0.36
## TotalSOD             26.60    165.57
## MnSOD                14.84    174.10
## CuZnSOD              32.73    198.99
## MalicAcid           -50.75     62.84
## SuccinicAcid        -20.31     16.98
## FumaricAcid           7.36     19.46
## Raffinose            -7.73     68.66
## Sucrose              -0.74      4.24
## Glucose               3.03      9.85
## Fructose             -9.31      9.62
## TotalSugars           2.58      1.97
## Scopoletin            1.33      0.86
## PolyPhenol          -17.72     28.31
## Ascorbic             15.01     12.51
## Ascorbate             0.86      1.22
## Guaiacol            -14.74     31.11
## Tocopherol           -4.34     10.25
## Proteins             -2.22      2.12
## ---
##   n = 60, k = 36
##   residual deviance = 21703.6, null deviance = 122979.3 (difference = 101275.7)
##   overdispersion parameter = 904.3
##   residual sd is sqrt(overdispersion) = 30.07
```

```
display(modellist[[3]])
```

```
## glm(formula = PPDscores ~ ., data = TabelaFinalTese2014[1:31])
##                  coef.est  coef.se  
## (Intercept)        1234.64    511.13
## SampleIAC             1.24    159.89
## SampleORI           -90.11    240.62
## SampleSAN           -93.52    271.83
## DaysDay3           -293.09    328.20
## DaysDay5           -128.66    211.58
## DaysDay8           -134.81    145.57
## DaysFresh          -424.97    368.41
## Phenolics            -0.28      0.17
## Flavonoids            0.03      0.04
## Carotenoids          -7.71      5.61
## Anthocyanins          0.89      1.85
## Totalcyanide      27608.13  12095.61
## AcetoneCyano     -27622.91  12099.96
## Linamarin        -27608.98  12095.58
## Linamarase          -53.52     56.65
## HydrogenPeroxide     -1.85      1.43
## Catalase             -0.14      0.36
## TotalSOD             26.60    165.57
## MnSOD                14.84    174.10
## CuZnSOD              32.73    198.99
## MalicAcid           -50.75     62.84
## SuccinicAcid        -20.31     16.98
## FumaricAcid           7.36     19.46
## Raffinose            -7.73     68.66
## Sucrose              -0.74      4.24
## Glucose               3.03      9.85
## Fructose             -9.31      9.62
## TotalSugars           2.58      1.97
## Scopoletin            1.33      0.86
## PolyPhenol          -17.72     28.31
## Ascorbic             15.01     12.51
## Ascorbate             0.86      1.22
## Guaiacol            -14.74     31.11
## Tocopherol           -4.34     10.25
## Proteins             -2.22      2.12
## ---
##   n = 60, k = 36
##   residual deviance = 21703.6, null deviance = 122979.3 (difference = 101275.7)
##   overdispersion parameter = 904.3
##   residual sd is sqrt(overdispersion) = 30.07
```

```
Model4 <- glm(PPDscores ~ ., data = secMetabo[-c(1:2)])
Model5 <- glm(PPDscores ~ ., data = Cyanogenic[-c(1:2)])
Model6 <- glm(PPDscores ~ ., data = Enzymes[-c(1:2)])
Model7 <- glm(PPDscores ~ ., data = SugarAcids[-c(1:2)])
Model8 <- glm(PPDscores ~ ., data = ROS[-c(1:2)])


display(Model4)
```

```
## glm(formula = PPDscores ~ ., data = secMetabo[-c(1:2)])
##              coef.est coef.se
## (Intercept)   2.21    20.49  
## Phenolics    -0.08     0.04  
## Flavonoids    0.04     0.01  
## Carotenoids   0.30     3.91  
## Anthocyanins  0.39     0.67  
## Scopoletin    0.18     0.12  
## ---
##   n = 60, k = 6
##   residual deviance = 93563.9, null deviance = 122979.3 (difference = 29415.5)
##   overdispersion parameter = 1732.7
##   residual sd is sqrt(overdispersion) = 41.63
```

```
AIC(Model4)
```

```
## [1] 625.3959
```

```
AIC(Model5)
```

```
## [1] 635.8171
```

```
AIC(Model6)
```

```
## [1] 610.141
```

```
AIC(Model7)
```

```
## [1] 593.961
```

```
AIC(Model8)
```

```
## [1] 612.7231
```

```
###using stargazer
require(stargazer)
summary(Model4)
```

```
## 
## Call:
## glm(formula = PPDscores ~ ., data = secMetabo[-c(1:2)])
## 
## Deviance Residuals: 
##     Min       1Q   Median       3Q      Max  
## -58.725  -31.577   -7.118   23.195  122.773  
## 
## Coefficients:
##              Estimate Std. Error t value Pr(>|t|)  
## (Intercept)   2.20652   20.49181   0.108   0.9147  
## Phenolics    -0.08297    0.03985  -2.082   0.0421 *
## Flavonoids    0.03714    0.01449   2.563   0.0132 *
## Carotenoids   0.30345    3.91011   0.078   0.9384  
## Anthocyanins  0.38901    0.67454   0.577   0.5665  
## Scopoletin    0.18222    0.11597   1.571   0.1220  
## ---
## Signif. codes:  0 '***' 0.001 '**' 0.01 '*' 0.05 '.' 0.1 ' ' 1
## 
## (Dispersion parameter for gaussian family taken to be 1732.664)
## 
##     Null deviance: 122979  on 59  degrees of freedom
## Residual deviance:  93564  on 54  degrees of freedom
## AIC: 625.4
## 
## Number of Fisher Scoring iterations: 2
```

```
summary(Model5)
```

```
## 
## Call:
## glm(formula = PPDscores ~ ., data = Cyanogenic[-c(1:2)])
## 
## Deviance Residuals: 
##     Min       1Q   Median       3Q      Max  
## -68.364  -39.798   -5.052   29.789  112.683  
## 
## Coefficients:
##                Estimate Std. Error t value Pr(>|t|)
## (Intercept)   1.388e+01  5.167e+01   0.269    0.789
## Totalcyanide  1.672e+04  1.449e+04   1.154    0.253
## AcetoneCyano -1.672e+04  1.449e+04  -1.154    0.253
## Linamarin    -1.672e+04  1.449e+04  -1.154    0.253
## Linamarase   -2.528e-01  5.758e+00  -0.044    0.965
## 
## (Dispersion parameter for gaussian family taken to be 2092.441)
## 
##     Null deviance: 122979  on 59  degrees of freedom
## Residual deviance: 115084  on 55  degrees of freedom
## AIC: 635.82
## 
## Number of Fisher Scoring iterations: 2
```

```
summary(Model6)
```

```
## 
## Call:
## glm(formula = PPDscores ~ ., data = Enzymes[-c(1:2)])
## 
## Deviance Residuals: 
##    Min      1Q  Median      3Q     Max  
## -70.49  -20.61  -10.76   25.66  106.78  
## 
## Coefficients:
##              Estimate Std. Error t value Pr(>|t|)    
## (Intercept) -13.15560   28.26173  -0.465 0.643487    
## PolyPhenol    1.48337    5.06397   0.293 0.770723    
## Ascorbic     10.16273    5.24657   1.937 0.058081 .  
## Ascorbate    -0.06942    0.23650  -0.294 0.770282    
## Guaiacol     16.22768    4.03651   4.020 0.000185 ***
## Tocopherol   -0.45231    3.25737  -0.139 0.890090    
## Proteins      0.32678    0.24160   1.353 0.181941    
## ---
## Signif. codes:  0 '***' 0.001 '**' 0.01 '*' 0.05 '.' 0.1 ' ' 1
## 
## (Dispersion parameter for gaussian family taken to be 1324.15)
## 
##     Null deviance: 122979  on 59  degrees of freedom
## Residual deviance:  70180  on 53  degrees of freedom
## AIC: 610.14
## 
## Number of Fisher Scoring iterations: 2
```

```
summary(Model7)
```

```
## 
## Call:
## glm(formula = PPDscores ~ ., data = SugarAcids[-c(1:2)])
## 
## Deviance Residuals: 
##     Min       1Q   Median       3Q      Max  
## -72.528  -21.402   -0.419   19.336   88.438  
## 
## Coefficients:
##              Estimate Std. Error t value Pr(>|t|)    
## (Intercept)   92.8606    19.1779   4.842 1.24e-05 ***
## MalicAcid     18.7041    18.1690   1.029  0.30813    
## SuccinicAcid  -6.9382     5.7054  -1.216  0.22955    
## FumaricAcid   -0.0505     3.1209  -0.016  0.98715    
## Raffinose    -15.6627     7.4786  -2.094  0.04122 *  
## Sucrose       -2.4380     0.9690  -2.516  0.01506 *  
## Glucose       -4.0160     1.2804  -3.136  0.00284 ** 
## Fructose      -0.3196     1.1682  -0.274  0.78553    
## TotalSugars    2.2975     0.9126   2.517  0.01500 *  
## ---
## Signif. codes:  0 '***' 0.001 '**' 0.01 '*' 0.05 '.' 0.1 ' ' 1
## 
## (Dispersion parameter for gaussian family taken to be 983.0493)
## 
##     Null deviance: 122979  on 59  degrees of freedom
## Residual deviance:  50136  on 51  degrees of freedom
## AIC: 593.96
## 
## Number of Fisher Scoring iterations: 2
```

```
summary(Model8)
```

```
## 
## Call:
## glm(formula = PPDscores ~ ., data = ROS[-c(1:2)])
## 
## Deviance Residuals: 
##     Min       1Q   Median       3Q      Max  
## -61.365  -25.507   -1.259   19.075  102.891  
## 
## Coefficients:
##                   Estimate Std. Error t value Pr(>|t|)    
## (Intercept)      -16.78133   15.43900  -1.087 0.281890    
## HydrogenPeroxide   0.38830    0.10912   3.558 0.000787 ***
## Catalase           0.10429    0.07686   1.357 0.180462    
## TotalSOD         -56.66691   70.33544  -0.806 0.423969    
## MnSOD             -3.56664   53.58815  -0.067 0.947181    
## CuZnSOD          143.25047   98.43518   1.455 0.151382    
## ---
## Signif. codes:  0 '***' 0.001 '**' 0.01 '*' 0.05 '.' 0.1 ' ' 1
## 
## (Dispersion parameter for gaussian family taken to be 1402.767)
## 
##     Null deviance: 122979  on 59  degrees of freedom
## Residual deviance:  75749  on 54  degrees of freedom
## AIC: 612.72
## 
## Number of Fisher Scoring iterations: 2
```

```
Table1B<-stargazer(Model4,Model5, Model6, Model7, Model8,Model1, type="text",Align=TRUE,
          title="Table 1B: Results of Ordinary Least Square (OLS) Regression models",
          intercept.bottom = FALSE,notes.label = "Significance levels:",
          dep.var.caption  = "OLS REGRESSION MODELS",
          dep.var.labels   = "DEPENDENT VARIABLE:PPD Scores",column.labels = c("Metabolites", "Cyanogenics","Enzymes","Sugar+Acids","ROS","All data"),
          model.numbers= FALSE,digits=1,cex=0.7)
```

```
## 
## Table 1B: Results of Ordinary Least Square (OLS) Regression models
## ==================================================================================
##                                          OLS REGRESSION MODELS                    
##                      -------------------------------------------------------------
##                                      DEPENDENT VARIABLE:PPD Scores                
##                      Metabolites Cyanogenics Enzymes Sugar+Acids  ROS    All data 
## ----------------------------------------------------------------------------------
## Constant                 2.2        13.9      -13.2    92.9***   -16.8    -191.1  
##                        (20.5)      (51.7)    (28.3)    (19.2)    (15.4)  (221.9)  
##                                                                                   
## Phenolics              -0.1**                                              -0.1   
##                        (0.04)                                             (0.1)   
##                                                                                   
## Flavonoids             0.04**                                              0.01   
##                        (0.01)                                             (0.03)  
##                                                                                   
## Carotenoids              0.3                                               -1.9   
##                         (3.9)                                             (5.1)   
##                                                                                   
## Anthocyanins             0.4                                               0.9    
##                         (0.7)                                             (1.5)   
##                                                                                   
## Scopoletin               0.2                                               0.4    
##                         (0.1)                                             (0.4)   
##                                                                                   
## Totalcyanide                      16,723.7                              20,536.5* 
##                                  (14,490.4)                             (11,608.2)
##                                                                                   
## AcetoneCyano                      -16,721.6                             -20,516.5*
##                                  (14,489.8)                             (11,607.2)
##                                                                                   
## Linamarin                         -16,723.2                             -20,535.1*
##                                  (14,490.3)                             (11,608.2)
##                                                                                   
## Linamarase                          -0.3                                   12.6   
##                                     (5.8)                                 (24.7)  
##                                                                                   
## PolyPhenol                                     1.5                        -18.9   
##                                               (5.1)                       (20.8)  
##                                                                                   
## Ascorbic                                      10.2*                        8.4    
##                                               (5.2)                       (11.7)  
##                                                                                   
## Ascorbate                                     -0.1                         -0.7   
##                                               (0.2)                       (0.9)   
##                                                                                   
## Guaiacol                                     16.2***                       11.7   
##                                               (4.0)                       (14.7)  
##                                                                                   
## Tocopherol                                    -0.5                         5.1    
##                                               (3.3)                       (6.4)   
##                                                                                   
## Proteins                                       0.3                         1.0    
##                                               (0.2)                       (1.3)   
##                                                                                   
## MalicAcid                                               18.7               20.5   
##                                                        (18.2)             (53.7)  
##                                                                                   
## SuccinicAcid                                            -6.9              -20.7   
##                                                         (5.7)             (14.0)  
##                                                                                   
## FumaricAcid                                             -0.1               4.8    
##                                                         (3.1)             (13.0)  
##                                                                                   
## Raffinose                                              -15.7**             40.2   
##                                                         (7.5)             (35.5)  
##                                                                                   
## Sucrose                                                -2.4**             -4.1**  
##                                                         (1.0)             (1.8)   
##                                                                                   
## Glucose                                                -4.0***             -2.6   
##                                                         (1.3)             (5.3)   
##                                                                                   
## Fructose                                                -0.3               -2.2   
##                                                         (1.2)             (5.1)   
##                                                                                   
## TotalSugars                                             2.3**              1.8*   
##                                                         (0.9)             (1.0)   
##                                                                                   
## HydrogenPeroxide                                                 0.4***    -0.1   
##                                                                  (0.1)    (0.4)   
##                                                                                   
## Catalase                                                          0.1      0.4    
##                                                                  (0.1)    (0.2)   
##                                                                                   
## TotalSOD                                                         -56.7    100.8   
##                                                                  (70.3)  (154.5)  
##                                                                                   
## MnSOD                                                             -3.6    -28.9   
##                                                                  (53.6)  (118.2)  
##                                                                                   
## CuZnSOD                                                          143.3    -50.2   
##                                                                  (98.4)  (177.6)  
##                                                                                   
## ----------------------------------------------------------------------------------
## Observations             60          60        60        60        60       60    
## Log Likelihood         -306.7      -312.9    -298.1    -288.0    -300.4   -274.3  
## Akaike Inf. Crit.       625.4       635.8     610.1     594.0    612.7    606.5   
## ==================================================================================
## Significance levels:                                   *p<0.1; **p<0.05; ***p<0.01
## 
## Table 1B: Results of Ordinary Least Square (OLS) Regression models
## ====
## TRUE
## ----
## 
## Table 1B: Results of Ordinary Least Square (OLS) Regression models
## ===
## 0.7
## ---
```

### correlation between variables with PPD are also provided as html document

### This document was produced by authors in R SOFTWARE to facilitate the reproduction of the experiment.
